# Global identification of the genetic networks and *cis*-regulatory elements of the cold response in zebrafish

**DOI:** 10.1093/nar/gkv780

**Published:** 2015-07-30

**Authors:** Peng Hu, Mingli Liu, Dong Zhang, Jinfeng Wang, Hongbo Niu, Yimeng Liu, Zhichao Wu, Bingshe Han, Wanying Zhai, Yu Shen, Liangbiao Chen

**Affiliations:** 1 Key Laboratory of Aquacultural Resources and Utilization, Ministry of Education, College of Fisheries and Life Sciences, Shanghai Ocean University, Shanghai 201306, China; 2 Institute of Genetics and Developmental Biology, Chinese Academy of Sciences, Beijing 100101, China

## Abstract

The transcriptional programs of ectothermic teleosts are directly influenced by water temperature. However, the *cis*- and *trans*-factors governing cold responses are not well characterized. We profiled transcriptional changes in eight zebrafish tissues exposed to mildly and severely cold temperatures using RNA-Seq. A total of 1943 differentially expressed genes (DEGs) were identified, from which 34 clusters representing distinct tissue and temperature response expression patterns were derived using the *k*-means fuzzy clustering algorithm. The promoter regions of the clustered DEGs that demonstrated strong co-regulation were analysed for enriched *cis*-regulatory elements with a motif discovery program, DREME. Seventeen motifs, ten known and seven novel, were identified, which covered 23% of the DEGs. Two motifs predicted to be the binding sites for the transcription factors Bcl6 and Jun, respectively, were chosen for experimental verification, and they demonstrated the expected cold-induced and cold-repressed patterns of gene regulation. Protein interaction modeling of the network components followed by experimental validation suggested that Jun physically interacts with Bcl6 and might be a hub factor that orchestrates the cold response in zebrafish. Thus, the methodology used and the regulatory networks uncovered in this study provide a foundation for exploring the mechanisms of cold adaptation in teleosts.

## INTRODUCTION

As teleost fishes are ectothermic, their body temperatures are generally identical to that of the external water. The environmental temperature thus exerts profound effects on the whole-body physiology of these fishes, from cardiovascular function to all aspects of locomotion (1). Temperature is also a key factor that limits the geographical distribution of fishes in nature; this distribution is largely determined by the capability of species’ genomic programs to adapt to temperature fluctuations. Eurythermal fishes can survive a broad range of temperature fluctuations, while stenothermal fishes cannot (2,3). Deciphering the genetic circuits that function in response to temperature fluctuations in distinct thermally adapted fishes is thus important for delineating the molecular mechanisms governing the adaptation and distribution of teleosts, the most widespread vertebrates in the world (4).

In the last decade, the genomic programs that respond to low-temperature challenge have been studied in both eurythermal and stenothermal fishes using genomic screening approaches. In one study, a time-course analysis of transcript expression in seven tissues was performed in eurythermal common carp (*Cyprinus carpio*) exposed to stepwise cooling regimes (5). This study showed that the expression levels of 3400 unique genes were altered during cold acclimation, and of them, 260 exhibited significantly and consistently altered expression levels in all seven tissues examined. A large number of genes were identified that exhibited highly tissue-specific expression patterns, suggesting that the cold response in this species is genetically organized into multiple tiers. Another study analysed transcriptomic changes in eurythermal killifish (*Austrofundulus limnaeus*) that were acclimated to three constant temperatures, ranging from 20 to 37°C, or exposed to daily temperature fluctuations of between 20 and 37°C (6). The authors identified large-scale changes in transcription and many key regulators of biological processes, such as cell growth and proliferation. The transcriptional changes appeared to be regulated by different genes for the constant and fluctuating temperature regimes. Most notably, the high-mobility group B1 (HMGB1) protein was postulated as a global gene expression-regulating temperature sensor. This possibility sheds new light on the regulatory circuits of gene expression that function in response to temperature in this fish. Transcriptome analysis has also been conducted on stenothermal fishes. For example, Chen *et al*. compared the tissue transcriptome profiles of the Antarctic notothenioid *Dissostichus mawsoni* with those of temperate teleosts, revealing differences in genetic programs that contribute to physiological fitness at different temperatures among the fishes (7), and these authors suggested the specific importance of proteomic homeostasis and anti-oxidation in the adaptation of Antarctic fishes to the constant cold environment. Another study of an Antarctic fish, *Pagothenia borchgrevinki*, revealed the essential roles of protein metabolism genes in physiological fitness following exposure to heat stress (8). These genomic studies have elucidated the global transcription patterns, Gene Ontology (GO) functional groups and potential regulators associated with cold stress responses (7,9–11). However, further studies on *trans-* and *cis-*elements that orchestrate cold-responsive pathways are needed to delineate the signaling pathways of the cold responses in teleosts.

Substantial progress has been made over the past decade in elucidating the transcriptional networks that regulate cold acclimation in plants. For example, the dehydration-responsive element/C-repeat (DRE/CRT) element, which is specifically recognized by C-repeat binding factors (CBFs), activates the transcription of cold-responsive genes in Arabidopsis (12,13). Gene fusion experiments and mutational analysis of the *CBF2* promoter have revealed two *cis*-elements, ICEr1 and ICEr2, that function in response to *CBF* expression during cold acclimation (14). These studies identified the detailed roles of *cis-*elements in regulatory networks, but such laborious perturbation analyses remain challenging. The recent availability of entire genome sequences for an increasing number of organisms should aid in achievement of this goal in a more efficient, high-throughput manner. Genome-wide scanning of upstream regions allows for rapid identification of *cis*-elements in sets of co-expressed genes (15–17), and studies have reported success in using this technique (18–20). The roles of *cis*-elements in fish regulatory networks are rarely explored and fish genomic resources are expanding, decoding of the regulatory networks of gene expression and their evolutionary patterns using genome-wide analysis is greatly anticipated.

One species of fish that has proven highly suitable for analysis of its regulatory program is the zebrafish, *Danio rerio*. This species has been a prominent model vertebrate in environmental biology. Several studies have documented changes in gene expression and related pathways in response to alterations in temperature in this fish (21–25). Two previous studies have shown that cold stress causes changes in the expression of several hundred genes during the early developmental stages (9,11). Zebrafish tolerates a wide range of temperatures, from 6.7 to 41.7°C, with optimal growth at 28°C (26,27). This species serves as a model that can be used to investigate the evolution of temperature-responsive networks by comparisons with fishes with narrower survival temperatures.

In this study, we conducted multi-tissue RNA-Seq on zebrafish exposed to increasing levels of cold. This comprehensive transcriptome analysis revealed common as well as tissue-specific gene expression patterns that arise in response to cold stress. Clusters of co-regulated genes were screened for over-represented *cis*-elements within their promoters using a motif prediction tool, DREME (28). We identified a series of potential motifs for the cold response in zebrafish, half of which are novel. A novel AGMAACCA motif and a known AP-1 motif were chosen for experimental validation and were confirmed to be *cis*-regulatory elements that drive target gene expression during cold exposure. The *trans*-factors that bind to these two motifs were determined to be Bcl6 and Jun, respectively, and they were shown to be interacting partners. Thus, genomics screening in combination with motif prediction can serve as a high-throughput approach to deciphering the molecular circuits that regulate environmental stresses.

## MATERIALS AND METHODS

### Fish and cold exposure

Six-month-old zebrafish were maintained at 28 ± 1°C. For cooling, the fish were subjected to a stepwise cooling process in increments of ∼0.85°C/h to either 18 or 10°C. Fish of both sexes were randomly assigned to each temperature. The brain, heart, liver, intestines, muscle, gill, spleen and kidneys were isolated. For each tissue type, tissues from 20 fish were combined to obtain an adequate amount of RNA for sequencing.

### RNA preparation

Total RNA was extracted using TRIZOL reagent according to the manufacturer's protocol (Invitrogen, Carlsbad, CA, USA). RNA degradation and contamination was monitored using 1% agarose gels. RNA quality was assessed by measuring the RNA integrity number (RIN) using a Bioanalyzer Chip RNA 7500 series II (Agilent, Santa Clara, CA, USA). Samples with an RIN value of greater with 8.0 were used for sequencing (Supplementary Figure S1). The total RNA concentration was determined with a Qubit fluorometer (Life Technologies).

### RNA sequencing

Three micrograms of RNA from each sample were used to prepare an mRNA-Seq library with a TruSeq RNA Sample Prep Kit (Illumina), following the manufacturer's instructions. Unique index codes were used to assign sequences to individual samples. Briefly, poly(A)^+^ RNA was purified and fragmented using divalent cations under elevated temperature conditions. RNA fragments were converted to cDNA using random primers, followed by second-strand cDNA synthesis and end repair. Illumina PE adaptors were attached to the cDNA ends. Fragments with an insert length of ∼300 bp were extracted from a 2% low-range ultra-agarose sizing gel. Adaptor-tagged cDNA fragments were enriched by 10-cycle PCR following the manufacturer's protocol. Library quality and insert length were checked using a High Sensitivity DNA Bioanalyzer Chip (Agilent) to ensure the proper insert size of 300–500 bp was attained (Supplementary Figure S2). The libraries were diluted to 10 pM, and equal amounts of 8 distinctively indexed libraries were mixed and subjected to 100 cycles of paired-end (2 × 100 bp) sequencing on one lane of an Illumina Hiseq 2000 system. A total of three lanes were used to sequence the entire sample set.

### Pre-processing and mapping of RNA-Seq reads

The quality of the raw reads was assessed using FASTX_toolkit (http://hannonlab.cshl.edu/fastx_toolkit). Reads for which 95% of the nucleotides had a Phred quality score of >5 were kept. To ensure the same order and integrity of the paired-end reads, a custom Perl script was utilized to filter two paired-end fastq files. The reference genome sequence and gtf files were downloaded from Ensembl release 72 (http://www.ensembl.org/info/data/ftp). TopHat (29) was used to map the reads to the reference genome; this mapping identified potential exons and novel splice junctions. We set the maximum intron size to 4 kb. The other TopHat parameters were set at the default settings for read mapping. The reads were mapped independently for each sample.

### Expression quantification and determination of differentially expressed genes

Samtools (30) was used to first sort the bam files containing the aligned reads from TopHat by read name. HTSeq-count (http://www.huber.embl.de/users/anders/HTSeq/) was then used to count the number of reads mapped to the genes. We next normalized gene expression using the reads per kilobase per million mapped reads (RPKM) methodology, in which the level of gene expression is evaluated according to the ratio between the number of mapped reads of the gene and the total number of mapped reads, taking into account the gene length (31). Genes with low read counts (<5 reads) were filtered out before gene expression analysis. Fisher's exact test was then used to identify DEGs between each pair of temperatures in each tissue, and Benjamini-Hochberg correction was performed. Genes with a fold change of >2 and an adjusted *P* value of <0.001 were considered differentially expressed. Multi-dimensional scaling (MDS) was performed using the function cmdscale with the parameter *k* = 2 in R. All statistical analyses were performed using R software.

### Validation of RNA-Seq results by reverse transcription quantitative real-time PCR (RT-qPCR)

Twelve genes were randomly selected for RT-qPCR analysis to evaluate the expression patterns deduced from the sequencing data. Two micrograms of RNA from each sample were reverse transcribed to cDNA using an RT-PCR kit (TaKaRa). qPCR was performed using SYBR Green Master Mix following the manufacturer's protocol (Roche). *β-Actin* was used as an internal control because the RNA-Seq data showed that its expression was relatively temperature independent. The primers used are listed in Supplementary Table S1.

### Cluster analysis

Genes that were differentially expressed between at least one pair of temperatures in any one of the eight tissues were considered cold responsive. The log_2_ fold changes in expression between the cold treatment and control for a total of 1943 cold-related genes were subjected to fuzzy *k*-means clustering (32). We set *k* = 60 for the clustering and then identified 34 centroids. We included genes with a significant membership value of 0.12, which was approximately five times the average value (1/35 = 0.028).

### GO enrichment analysis

To identify GO functional categories enriched under cold stress, GO annotation files were downloaded from the Ensembl database. The R package GOseq (33), which accounts for length bias, was used to perform enrichment analysis. GO categories that were enriched, as determined using a cut-off false discovery rate (FDR) of 0.05, were retained for further analysis.

### Motif analysis

One kilobase-long DNA sequences upstream of the annotated 5′ UTRs of the genes were retrieved using BioMart from Ensembl release 72. Overrepresented motifs in the upstream sequences were identified using DREME (28), which is available as part of MEME Suite of motif-based sequence analysis tools (http://meme.nbcr.net). DREME computes the (uncorrected) *P* value of Fisher's exact test to determine the significance of the representation of each motif in the positive set compared with that in the negative set. For each motif, the *E*-value was calculated with the corrected *P* value. Motifs with an *E*-value of <0.05 were considered significantly enriched. The upstream sequences of the genes with <2-fold RPKM differences in any one of the three comparisons were used as a negative set and assigned the ‘-n’ option. We then aligned those enriched motifs to sequences in the JASPAR database using TOMTOM with a *P* value cut-off of 5e−3, and sequences with differences of <2 bases compared with known motifs were retained.

### Vector construction for cell transfection and zebrafish transgenic study

To validate the roles of the identified motifs, upstream promoter sequences containing the selected motifs with the proper lengths from six genes (*gstp1, c1orf51, fosl1a, ubc, serpina7* and *apoba*) were PCR amplified from zebrafish genomic DNA. For cell transfection, the tested promoter sequences were cloned into a PGL4.10 vector. Motif mutants were created using a Q5 Site-Directed Mutagenesis Kit (NEB) by replacing the nucleotides of the wild-type motif with an equal number of thymidines in the promoter sequence.

The wild-type and mutant promoter sequences were cloned into a Tol2 transposable element-based transgenic vector, which was modified from pEGFP-N1 (CLONTECH). The left and right ends of the Tol2 element were PCR amplified from medaka genomic DNA according to the published sequence (accession no. D84375) (34). The right end was inserted into pEGFP-N1 at the AflII site, followed by the SV40 polyA signal. The left end was inserted between the AseI and NheI restriction sites, replacing the original cauliflower mosaic virus (CMV) promoter. The resulting plasmid contained a multiple cloning sequence (MCS) followed by an *egfp* coding sequence, flanked by the arms of the Tol2 transposon. The promoter sequences for testing were cloned into the XhoI and BamHI sites within the MCS to drive *egfp* expression. The sequences of the constructed plasmids were verified by DNA sequencing (3730XL, Applied Biosystems). The primers used for plasmid construction are listed in Supplementary Table S1.

### Cell culture, transient transfection, cold treatment and RT-qPCR analysis

ZF4 cells (ATCC) were maintained in DMEM/F12 medium with 10% FBS at 28°C. The CIK cell line, derived from the kidney of grass carp (*Ctenopharyngodon idellus*), was maintained in DMEM/F12 medium with 10% FBS at 28°C (35). Plasmid DNAs were transiently transfected into ZF4 cells or CIK cells using Fugene HD (Promega) following the manufacturer's protocol. Briefly, 0.8 μg of PGL4 plasmid carrying the upstream motif sequence or the mutated motif sequence and 0.2 μg of a control pRL-CMV plasmid were transfected into 1.5 × 10^5^ cells in a 6-well plate. Forty-eight hours after transfection, the cells were transferred to a low-temperature incubator (Galaxy 170R, Eppendorf) and exposed to a temperature of 10°C for 24 h. After the cold treatment, cells at 28°C and 10°C were harvested for total RNA isolation. RT-qPCR was performed as described above. The expression levels of firefly luciferase were normalized to those of Renilla luciferase. All assays were conducted in three parallel experiments and repeated three times. The primers used for qPCR are listed in Supplementary Table S1.

### *In vivo* validation of cold-responsive functions of *cis*-motifs

In each transgenic experiment, over 300 single-cell zebrafish embryos from one parent were equally divided, and constructed Tol2 vectors containing the wild-type or mutant promoter (50 ng/μl) together with 50 ng/μl transposase mRNA were microinjected separately into the eggs. The injected embryos were screened for GFP expression at 48 h post-fertilization (hpf). GFP-positive embryos were imaged live using a Carl Zeiss SteREO Discovery V20 microscope. Half of the GFP-positive embryos were randomly selected for exposure to low temperature (10°C), and the remaining GFP-positive embryos were incubated at the control temperature (28°C). Embryos were collected for RNA extraction after 12 h of treatment. Equal amounts of total RNA from similar numbers of embryos with the wild-type and mutant constructs were analysed by RT-qPCR to determine *egfp* expression, and *β-actin* was used as a control. The cold-induced fold change in the *egfp* mRNA level at 10°C was normalized to that at 28°C. The primers used are listed in Supplementary Table S1. At least three replicate experiments were performed for each construct.

### Co-immunoprecipitation and western blot

ZF4 cells were cold treated as described above (see cold treatment). Cells maintained at 28 and 10°C were harvested and lysed in NP40 buffer (50 mM Tris, 50 mM NaCl, 0.1% Triton X-100, 1% Nonidet P-40 and protease inhibitor cocktail (Sigma)) at 4°C. The supernatant was obtained by centrifugation at 4°C (12 000 g, 20 min). The Bradford method (Bio-Rad) was used to determine the protein content. After pre-clearing with protein A/G agarose beads (Pierce, #20421), 500 μg of cell lysate protein was incubated with 4 μl of an anti-Bcl6 antibody (Abcam, ab86861) or 8 μl of an anti-Jun antibody (Abcam, ab31419) at 4°C overnight prior to incubation for 6 h with 60 μl protein A/G agarose beads. A control reaction was performed in parallel by replacing the antibody with normal rabbit IgG (Santa Cruz, sc-2027). The immune complexes were pelleted at 100 g for 20 min, and the supernatant was removed by aspiration. The complexes were then washed 6 times with ice-cold lysis buffer. Next, 30 μl of 4× loading buffer (TaKara, #9173) was added to the complexes, and the samples were boiled for 10 min. The samples were electrophoresed on 12% SDS-polyacrylamide gels and electro-transferred to PVDF membranes. The membranes were incubated with an anti-Bcl6 (1:1000) or anti-Jun (1:1000) antibody. A VeriBlot for IP secondary antibody (1:1000, Abcam, ab131366) was used to reduce interference from the IP antibody. The colour was developed after incubation with the secondary antibody using an ECL kit (Bio-Rad, #1705061) according to the manufacturer's instructions.

## RESULTS

### Analysis of cold-induced gene expression patterns in zebrafish tissues

**Figure 1.**
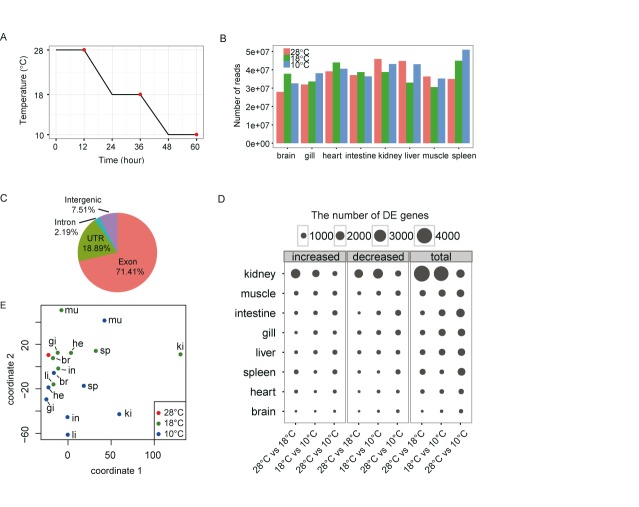
Characterization of the cold-induced gene expression patterns in zebrafish tissues. (**A**) Schematic diagram showing the cooling time course and the sampling regimes. The fish were subjected to the following three temperatures: 28, 18 and 10°C. The red dots indicate the sampling time points. At each point, 20 fish were sampled, and RNA from eight tissues was isolated and pooled by tissue type for RNA-Seq. (**B**) The number of RNA-Seq reads obtained from the eight analysed tissues at the three temperatures. (**C**) The read distributions among the annotated zebrafish genomic features. (**D**) The tissue distributions of DEGs with a >2-fold RPKM difference for the three comparisons (28°C compared with 18, 18°C compared with 10 and 28°C compared with 10°C). The sizes of the circles are proportional to the numbers of genes that they contain. (**E**) MDS plot showing the diversity of the responses to the cold treatment among the zebrafish tissues. The entire set of expressed genes (22 457 genes) was used in analysis. The MDS algorithm placed each sample in a 2-dimensional space for visualization of the level of similarity of the tissue expression patterns. Each sample was assigned coordinates based on the expression levels of all genes in the sample, and these coordinates were used to construct a scatterplot. The three temperatures are indicated by different colours. Abbreviations: br for brain, gi for gill, he for heart, in for intestine, ki for kidney, li for liver, mu for muscle and sp for spleen.

To obtain a comprehensive view of the transcriptional adaptation to different levels of cold stress, we treated 60 zebrafish at 6 months of age with a stepwise temperature decline from 28°C to 18°C and then to 10°C (Figure 1A). Twenty fish, with equal numbers of female and male individuals, were sampled after being maintained for 12 h at the desired temperature. Eight tissues, including brain, heart, gill, intestine, kidney, muscle, liver and spleen tissues, were collected from the individuals and pooled by the tissue type and sampling temperature to extract an adequate amount of total RNA for RNA-Seq. Using paired-end sequencing, we generated a total of 298.1, 301.3 and 319.9 million 100-bp paired-end reads for the 28, 18 and 10°C samples, respectively. The details for the individual tissues are shown in Figure 1B. The number of reads obtained for each sample ranged from 27.9 to 50.8 million. Using TopHat mapping (36), ∼85% of the clean reads were matched with the reference zebrafish genome (Supplementary Table S2), and nearly 90% (71.4% exon and 18.9% UTR sequences) of them were mapped to the annotated zebrafish reference genome (Ensembl release 72) (Figure 1C), indicating adequate representation of the sequenced genes within the genome.

Next, we investigated the gene expression changes among three pairs of temperatures (28°C compared with 18°C, 18°C compared with 10°C and 28°C compared with 10°C). We identified genes with >2-fold differences in the RPKM values for the three comparisons. The tissue distributions and directions of the expression changes (up- or down-regulation) of these DEGs are shown in Figure 1D and Supplementary Figure S3A. The tissues varied substantially in the numbers of DEGs. The kidney showed the largest number of DEGs, with 4143, 3792 and 1859 at 28°C compared with 18°C, 18°C compared with 10°C and 28°C compared with 10°C, respectively. In contrast, only 146, 314 and 573 DEGs, respectively, were identified in the brain. The tissue types listed in decreasing order according to the number of DEGs detected are as follows: kidney, muscle, intestine, gill, liver, spleen, heart and brain (Figure 1D). Interestingly, the number of DEGs identified at 28°C compared with that at 10°C was far less than that observed at either 28°C compared with 18°C or 18°C compared with 10°C in the kidney (Figure 1D). This finding was due to the presence of large numbers of genes with opposing expression changes under the different temperature challenges (18°C, 10°C). The changes in expression at 18°C were more divergent than those at 10°C (Supplementary Figure S3B). These results indicate that gene expression is a dynamic process that depends on multiple factors, such as the severity of the cold temperature and the tissue type. Overall, the number of up-regulated genes was nearly equal to that of down-regulated genes in almost all of the tissues (Figure 1D). We used different stringency levels to screen for DEGs, and the kidney always showed the highest number of DEGs in this study. In teleosts, the kidney is involved in many critical functions, such as haematopoiesis, immunity and excretion (37). The high percentage of cold-regulated genes in this organ suggests that these functions play important roles during cold stress.

To further examine the tissue-related cold-responsive patterns, we performed MDS analysis of the gene expression patterns in the eight tissues under the cold treatments. We first log_2_ transformed the datasets and normalized the gene expression at the low temperatures (18°C and 10°C) to that at the normal temperature (28°C). For each tissue, the expression of all of the genes at 28°C was normalized to the same value of 0, which is indicated by a red dot on the MDS plot. The degree of cold-induced changes in expression in each individual tissue could be inferred by the distance to the red dot. We observed temperature-dependent and tissue-dependent cold response patterns in the plot (Figure 1E). Generally, a lower temperature (10°C) induced stronger expression changes than a milder temperature (18°C) in all tissues except for the kidney. The kidney, liver and intestine showed more extensive expression alterations compared with the other tissues at both temperatures.

As a justification for the choice of 12 h intervals for RNA sampling in the cold temperature treatment, we evaluated the effect of a prolonged period (36 h) of cold exposure on the changes in the gene expression patterns in the fish. We reduced the temperature of the zebrafish from the normal temperature of 28–18°C, as was performed for the cold treatment in the previous experiment, and we maintained the fish at 18°C for 36 h prior to obtaining samples for RNA isolation (Supplementary Figure S3C). Five DEGs, *atf4b2, bcl2, bzw1b, cirbp* and *id1*, were chosen to examine the expression differences between 12 and 36 h at 18°C by RT-qPCR. All 5 genes were strongly induced in almost all of the tissues at 12 h compared with their expression in the control samples (28°C). By contrast, the expression of each gene remained largely unchanged from 12 to 36 h during exposure to the same cold temperature (18°C) (Supplementary Figure S3D). Cold exposure for 12 h appeared to be sufficient for promoting expression of the cold-responsive genes, particularly the early responsive genes. To a lesser extent, some cold-responsive genes were likely not detected between the sampling intervals due to the dynamic nature of gene expression during cold adaptation. This possibility could be reduced by performing sampling more frequently over the course of cold treatment.

**Figure 2.**
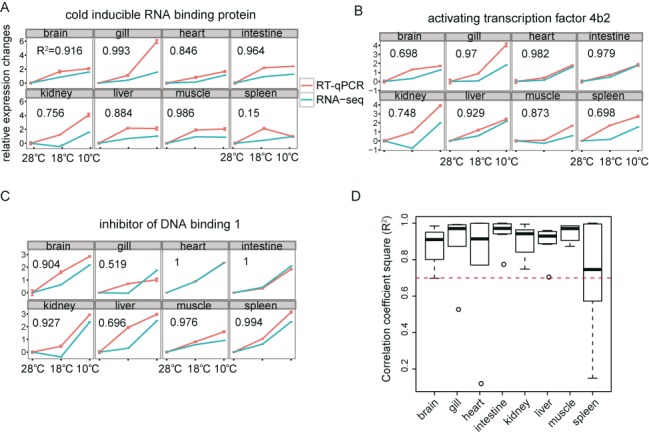
Comparison of the expression patterns of selected genes detected in RNA-Seq and RT-qPCR assays showing high correlation between the two methods. The log_2_ ratios of the expression changes at the low temperatures relative to the normal temperature were calculated and plotted (the ratio was set to 0 for the normal condition). The red line depicts the RT-qPCR results. The expression levels of the selected genes were normalized against that of *β-actin*. The blue line shows the RNA-Seq results. The *R*^2^ values (Pearson correlation coefficients) across the different temperatures are shown for each gene. The expression patterns of three genes—*cirbp* (**A**), *atf4b2* (**B**) and *id1* (**C**)—showed excellent agreement between the RNA-Seq and RT-qPCR assays. A boxplot of *R*^2^ values for the 12 selected genes shows a high *R*^2^ value (>0.7) across the tissues examined (**D**). Supplementary Figures S4 shows 9 additional genes that were also verified.

To validate the expression profiles identified with RNA-Seq, the relative mRNA levels for 12 genes across all eight (or fewer) tissues were measured by qPCR (Supplementary Figure S4). The correlation between the RNA-Seq and RT-qPCR results was analysed by calculating the coefficient of determination (*R*^2^). The expression data for three selected genes, *cirbp, atf4b2* and *id1*, in the eight tissues showed excellent agreement between RNA-Seq and RT-qPCR (Figure 2A, B and C). Although the expression data for a few genes, for example, those of *cirbp* in the spleen, were associated with slightly low *R*^2^ values across the temperatures tested, the trend of up-regulation at 10°C was consistent between these two tests. We plotted the *R*^2^ values for a total of 12 genes in the eight tissues (Figure 2D), and the high *R*^2^ value (>0.7) determined for the correlation between RT-qPCR and RNA-Seq data confirmed the reliability of differential expression analysis performed in this study.

### Gene clustering analysis revealed common and tissue-specific expression patterns

We observed the complex transcriptional changes that occurred under cold stress in multiple tissues. To identify co-regulated genes, we clustered the DEGs using the fuzzy *k*-means clustering method. To reduce noise, we screened the abovementioned DEGs using higher stringency. Genes that simultaneously satisfied two criteria, i.e. a >2-fold RPKM change and an FDR of <0.1% in at least one comparison by Fisher's exact test, were selected to generate a more compact set of DEGs. A total of 1943 genes were included in this set (Supplementary Table S3). The fuzzy k-means clustering method is widely used to cluster complex datasets and has been demonstrated to produce biologically relevant results (17,32). This method allows a single gene to be clustered into different groups and thus accommodates multiple biological functions, and it requires no *a priori* information about the dataset (32). Out of the 1943 genes, 1807 exhibited a significant membership value in 34 clusters (membership value > 0.12) (Supplementary Table S4). The number of genes and the expression change patterns for the 34 clusters are shown in Table 1 and Supplementary Figure S5.

**Table 1.** Summary of 34 clusters of differentially expressed genes

Cluster ID	Genes	Pattern	Temperature	Tissue
Cluster 0	231	Commonly induced	18°C, 10°C	all
Cluster 1	121	Commonly repressed	18°C, 10°C	all
Cluster 2	33	Induced in muscle; repressed in gill, heart, kidney, liver and spleen	18°C, 10°C	muscle, gill, heart, kidney, liver and spleen
Cluster 3	98	Repressed in liver, particularly at 18°C	18°C	liver
Cluster 4	73	Induced in kidney, particularly at 18°C	18°C	kidney
Cluster 5	71	Repressed in gill; induced in muscle, liver and spleen	18°C, 10°C	gill, muscle, liver and spleen
Cluster 6	8	Too few members		
Cluster 7	272	Except gill and heart at 10°C, induced in all tissues, especially in muscle	18°C, 10°C	all
Cluster 8	1	Too few members		
Cluster 9	13	Too few members		
Cluster 10	3	Too few members		
Cluster 11	52	Repressed in gill, kidney and liver at 18°C	18°C	gill, kidney, heart
Cluster 12	177	Induced in all tissues at 10°C, except muscle	10°C	all
Cluster 13	21	Too few members		
Cluster 14	10	Too few members		
Cluster 15	24	Too few members		
Cluster 16	128	Repressed in muscle	18°C, 10°C	muscle
Cluster 17	93	Repressed in all tissues but irregular in liver	18°C, 10°C	all
Cluster 18	84	Repressed in kidney but induced in liver	18°C, 10°C	kidney and liver
Cluster 19	188	Repressed in kidney	18°C, 10°C	kidney
Cluster 20	182	Repressed in gill, kidney and liver at 18°C	18°C	gill, kidney and liver
Cluster 21	72	Induced in intestine, kidney and spleen	18°C, 10°C	intestine, kidney and liver
Cluster 22	48	Mainly repressed in all tissues	18°C, 10°C	all
Cluster 23	20	Too few members		
Cluster 24	64	Repressed in gill and liver	18°C, 10°C	gill and liver
Cluster 25	55	Repressed in spleen	18°C, 10°C	spleen
Cluster 26	49	Repressed in gill and kidney	18°C, 10°C	gill and kidney
Cluster 27	29	Too few members		
Cluster 28	71	Induced in intestine, kidney, liver and spleen	18°C, 10°C	intestine, kidney, liver and spleen
Cluster 29	27	Too few members		
Cluster 30	57	Repressed in gill, intestine, kidney and spleen but induced in liver	18°C, 10°C	gill, intestine, kidney, spleen and liver
Cluster 31	51	Mixed response: induced in heart, muscle, intestine, liver and spleen	18°C, 10°C	heart, muscle, intestine, liver and spleen
Cluster 32	28	Too few members		
Cluster 33	38	Mixed response: initially induced but then repressed in gill, heart, kidney and spleen	18°C, 10°C	gill, heart, kidney and spleen

The six most populated clusters, Clusters 0, 1, 7, 12, 16 and 19, contained 231, 121, 272, 177, 128 and 188 genes, respectively, for a total of 1015 genes, which accounted for 56% of the DEGs. In contrast, Clusters 6, 8, 9, 10, 13, 14, 15, 23, 27, 29 and 32 each contained fewer than 30 members; thus, no general cold-response trend could be reliably deduced. Characteristic patterns could be assigned to the other clusters according to either the temperature(s) or the tissue(s) with which they were associated (Table 1). For example, a few clusters (3, 11, 12 and 20) contained genes with expression changes between only one temperature pair (either between 28 and 18°C or between 18 and 10°C), whereas most of the clusters contained genes with altered expression between both of these temperature pairs. Tissue specificity was more subtly distinguished among the clusters; six clusters (0, 1, 7, 12, 17 and 22) contained genes showing differential expression in all eight tissues, and the expression changes of genes in the other clusters were confined to a variable number of tissues, ranging from 6 (broad) to 1 (specific). These results indicated that fuzzy *k*-means clustering was sensitive for identifying common and tissue-specific expression patterns under mixed conditions. These clustered genes form a foundation for further functional and regulatory network studies of the cold response in zebrafish.

### GO terms over-represented in the zebrafish cold response

**Figure 3.**
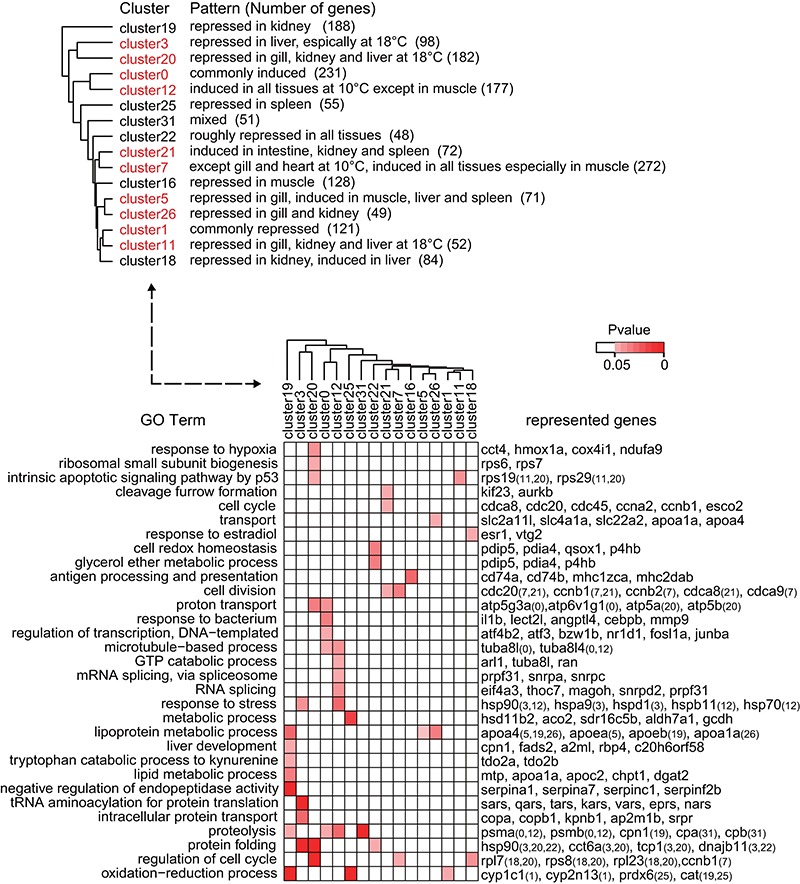
Heatmap showing the enriched GO categories within the gene clusters. All clusters listed in Supplementary Table S3 were examined for overrepresented GO terms by comparisons with the entire expressed gene set. Enriched GO terms were identified for the 16 clusters shown at the top. The associated biological process GO terms identified in each cluster are plotted in the bottom heatmap panel. The genes annotated with each enriched GO term are shown to the right of the panel. For enriched GO terms associated with genes from two or more clusters, the cluster(s) to which the genes belong are denoted by Cluster IDs in brackets. The color scales depict the p values for the enrichment tests, and the grey cells indicate a *P* value of >0.05. The results of the GO enrichment test are shown in Supplementary Table S5.

We performed GO enrichment analysis to reveal the over-represented biological processes associated with the clusters using GOseq (33). We identified 31 non-redundant enriched GO terms for biological processes from 16 clusters (Figure 3 and Supplementary Table S5 for all GO terms identified). We then grouped the expression clusters according to the distribution of enriched GO terms in each cluster. We found that clusters with similar expression patterns were grouped together and that they shared the same or similar GO terms. These clusters were clearly separable from the other patterns (Figure 3). For example, the genes in the closely grouped Clusters 3 and 20 were repressed in the liver, whereas those in Clusters 0 and 12 were induced in almost all of the tissues examined (Table 1).

We identified five enriched GO categories associated with the 231 commonly induced genes in Cluster 0. Many of the enriched terms and their associated genes have been reported to be involved in the cold response in fishes in other studies. For example, *bzw1b, nrf1d*, and *fosl1a* are involved in transcriptional regulation; *tuba8l* and *tuba8l4* are involved in microtubule-based processes; *prpf31, snrpa* and *snrpc* are involved in mRNA splicing; and the *psma, psmb* and *psmd* proteasomal subunits are involved in proteolysis. These example genes have also been shown to be up-regulated in common carp (5) and larval zebrafish (9,11) during cold challenges. However, we identified transcription factors that have not been previously reported to be responsive to cold in any fish. For example, *atf4b2*, which had the highest membership value (0.965) in Cluster 0 (Supplementary Table S4), has been previously reported to play roles in the adaptive response to cold stress only in hibernating ground squirrels (38) and mice (39), in which *atf4* activates downstream genes associated with energy homeostasis. Thus, this newly identified transcription factor may open new avenues for investigation of the regulatory network for cold stress in zebrafish.

In contrast with the existence of multiple enriched GO terms in the commonly induced gene cluster, only one term, the oxidation-reduction process, was found to be over-represented in the commonly repressed cluster (Cluster 1, 121 genes). This finding may reflect the overall slowing of biological reactions across all of the tissues at the cold temperatures. Enrichment of the oxidation-reduction GO term was only identified in two other repressed gene clusters, Clusters 19 and 25, which are specific to the kidney and spleen, respectively (Figure 3).

The enriched GO categories associated with tissue-specific clusters more or less reflected the cold-responsive biological processes confined to certain tissue types. For example, Cluster 3, which comprised 98 genes that were down-regulated in the liver, was enriched with aminoacyl-tRNA synthetases (aaRSs), including *qars, vars, eprs, tars, kars*, and *sars*, which are involved in tRNA aminoacylation for protein translation (Figure 3). aaRSs are critical for translation and play an essential role in protein synthesis (40). The levels of specific aaRSs affect the homeostasis of specific proteins, which could lead to long-term liver damage at severely cold temperatures. We also found GO terms and gene groups that were indicative of drastic cold stress responses in the gill and kidney. The genes in Cluster 26 were repressed in the gill and kidney and included those encoding 3 solute carrier family transporters (*slc2a11l, slc4a1a* and *slc22a2*), which are responsible for ion transport, and 2 encoding apolipoproteins (*apoa1a* and *apoa4*), which are associated with cholesterol efflux, suggesting the particular importance of ion transport and lipid metabolism in the cold response in these two tissues. Notably, the down-regulation of many genes encoding serine protease inhibitors (*serpins: serpina1, serpina1l, serpina7* and *serpinc1*) was also observed in the kidney. Cold decreases the rate of circulation and increases the viscosity of the blood, and both of these changes lead to an increased risk of clotting. Fishes inhabiting a constant cold environment, such as notothenioids in the Antarctic, counteract this effect by drastically reducing the number of circulating erythrocytes to reduce blood viscosity (41), indicating the critical importance of anti-clotting in the cold adaptation of teleosts. Serpins, such as Serpinc1, inhibit thrombin, thereby blocking activation of the feedback loop for blood coagulation (42). Hibernating turtles have been found to counteract the potential for cold-induced blood coagulation by up-regulating *serpins* (43). The widespread down-regulation of *serpins* in the zebrafish kidney may be indicative of a vulnerable point in the genetic circuits that hinders the tolerance of this fish to more severe cold temperatures.

### Identification and analysis of *cis*-regulatory elements in zebrafish cold response

**Figure 4.**
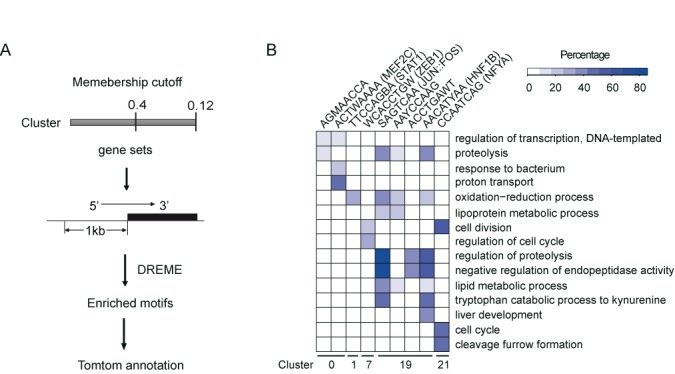
Identification of cold-responsive *cis*-regulatory motifs and distribution of the motif-containing genes among the enriched GO terms. (**A**) Strategies for identifying enriched motifs in the proximal promoter regions of the co-regulated genes. Genes were assigned to a cluster at two different membership cut-off values—0.12 and 0.4—based on the Pearson correlation of the gene's expression pattern with the centroid of the cluster. Conserved motifs that were within 1 kb upstream of their annotated 5′ UTRs were identified using the DREME algorithm. TOMTOM was used to annotate the detected motifs with JASPAR database. (**B**) Heatmap showing the percentages of genes in the gene set with certain motifs (shown at the top) associated with the enriched GO terms (shown at right) identified in Figure 3.

Genes exhibiting similar expression patterns are likely to be co-regulated at the transcriptional level through programmed interactions between *cis*-regulatory elements and *trans*-factors (e.g., TFs). The gene clusters generated in the abovementioned experiment allowed for us to identify the *cis*-regulatory elements involved in the cold response and adaptation in zebrafish. We screened for enriched nucleotide motifs (potential *cis*-regulatory elements) within the regions 1 kb upstream of the annotated 5′ UTRs of the clustered genes using the motif-finding algorithm DREME (28). The workflow is shown in Figure 4A. We initially searched from 500 bp to 5 kb of the upstream regions to detect enriched motifs, and we eventually decided to search the 1 kb upstream regions to balance detection sensitivity and the breadth of motif coverage. The motifs identified in this study are considered to be the proximal regulatory elements of the genes. The p values for the identified motifs were calculated based on a hypergeometric distribution, and those with a corrected p value (E-value) of lower than 0.05 were regarded as significantly over-represented motifs in their respective cluster; thus, these motifs may participate in a regulatory network that orchestrates similar expression patterns among the motif-bearing genes upon cold challenge. We identified 17 motifs from 7 clusters (Clusters 0, 1, 5, 7, 16, 19 and 21) (Table 2). No enriched motifs were identified in the genes in any of the other clusters. Among the clusters that yielded enriched motifs Clusters 19 and 7 contained the greatest numbers of motifs (5 and 4, respectively), with the others contained 1 or 2 enriched *cis*-motifs (Table 2). The over-represented motifs were compared with known motifs in JASPAR database using TOMTOM tool (44). Of the 17 enriched motifs, 10 had identical sequences as those of known transcription factor binding sites in JASPAR database; thus, they were regarded as known motifs (Table 2 and Supplementary Figure S6). The percentage of genes within each cluster with a specific motif varied. Cluster 7 contained the highest proportion of genes with the WCACCTGW motif (48%), suggesting that approximately half of the co-expressed genes in this cluster might be influenced by this motif.

**Table 2.** Enriched motifs in different clusters

Cluster ID	Membership cut-off	Enriched motif	Positive	Negative	*P*-value	*E*-value	Annotated name (ID)
Cluster 0	0.12	AGMAACCA	39/230	1478/20474	7.20E-07	3.10E-02	Novel
Cluster 0	0.4	ACTWAAAA	36/68	4894/20474	2.50E-07	7.90E-03	Novel
Cluster 1	0.12	CGCCCCW	25/121	1352/20474	3.50E-07	1.30E-02	Novel
Cluster 1	0.12	TTCCAGBA	34/121	2376/20474	7.30E-07	2.70E-02	Stat1(MA0137.3)
Cluster 5	0.4	GATAASAC	8/22	778/20474	8.90E-07	1.90E-02	Gata1(MA0037.2)
Cluster 7	0.12	WCACCTGW	113/272	3315/20474	6.80E-23	2.80E-19	Zeb1(MA0103.2)
Cluster 7	0.12	GCGGCCTA	15/272	49/20474	3.50E-15	1.50E-02	Novel
Cluster 7	0.4	ATTGGTCY	33/205	815/20474	1.60E-11	6.30E-07	Novel
Cluster 7	0.12	ACCAAYYG	59/272	1493/20474	6.80E-14	2.80E-09	Nobox(MA0125.1)
Cluster 16	0.12	BCCTTATA	24/128	1166/20474	2.80E-07	1.00E-02	Srf(MA0083.2)
Cluster 19	0.12	SAGTCAA	80/188	4765/20474	4.80E-09	1.90E-04	Jun::Fos(MA0099.2)
Cluster 19	0.12	AAYCCAAG	25/188	863/20474	5.40E-07	2.10E-02	Novel
Cluster 19	0.4	AACATYAA	55/118	4736/20474	2.10E-08	7.60E-04	Hnf1b(MA0153.1)
Cluster 19	0.4	AACTGWCC	30/118	1967/20474	6.20E-07	2.20E-02	Myb(MA0100.2)
Cluster 19	0.4	ACCTGAWT	24/118	1331/20474	6.10E-07	2.20E-02	Novel
Cluster 21	0.12	CCAATCAG	20/72	1590/20474	4.00E-07	1.30E-02	Nfya(MA0060.2)
Cluster 21	0.12	AAACCGCG	8/72	210/20474	9.40E-07	3.10E-02	Runx1(MA0002.2)

‘Positive’ indicates the number of genes that contain the enriched motif in the cluster, whereas ‘Negative’ indicates the number of genes that contain the enriched motif but are not differentially expressed in the entire gene set (see ‘Materials and Methods’ section). One gene in Cluster 0 was excluded from statistical analysis because of a lack of sequence information for the promoter region. The annotated names and IDs are shown according to JASPAR database. M, A or C; W, A or T; B, T, C or G; S, C or G; Y, C or T.

To reveal which GO terms might be regulated by the identified *cis*-motifs, we compared the GO terms associated with the motif-bearing genes (Supplementary Table S6) to those assigned to all of the genes within the cluster. The association matrix between the enriched motifs and the over-represented GO terms is shown in Figure 4B and Supplementary Table S7. A total of 15 GO terms were found to be associated with the enriched *cis*-motifs. These GO terms comprised 48% (15/31) of the total GO terms that were over-represented for the whole set of DEGs in the cold-stressed zebrafish (Figure 3), suggesting that the *cis*-elements identified by this method may participate in the transcriptional regulation of approximately half of the cold-responsive processes in this fish.

Previous studies have provided the first evidence of transcriptional regulation mediated by the identified motifs. For example, the TGAGTCA motif identified in genes in Cluster 19 is a known binding site for activator protein-1 (AP-1), which is a family of dimeric basic region-leucine zipper (bZIP) proteins that includes the Jun and Fos sub-families (45,46). A repressive role of the AP-1 binding site in *serpinb2* transcription has been recently identified (47). Consistently, this motif is carried by 87% (6/7) of the genes involved in the ‘negative regulation of endopeptidase activity’ (Figure 4B), including five *serpins* in zebrafish. The binding of c-Jun and JunB to the AP-1 site has been found to negatively regulate apolipoprotein M (*apom*) promoter activity (48), and we found that 4 apolipoprotein genes (*apoa2, apoba, apoc2*, and *apoeb*) containing this motif were repressed in the kidney (Figure 3). Another motif, the TTCCAGBA motif, detected in genes in Cluster 1, was identified as a Stat1 binding site and was over-represented in genes involved in the oxidation-reduction process (Figure 4B), in agreement with the known role of Stat1 as a transcriptional modulator of the genes responsible for energy metabolism pathways (49). Stat1 has also been reported to be involved in the lipopolysaccharide-mediated modulation of two cytochrome P450 enzymes (CYP2E1 and CYP1A) in the mouse liver (50), consistent with our finding that *cyp1c1* in zebrafish, which contains the Stat1 site, was repressed in Cluster 1 (Figure 3).

### Experimental validation of the *cis*-regulatory motifs

**Figure 5.**
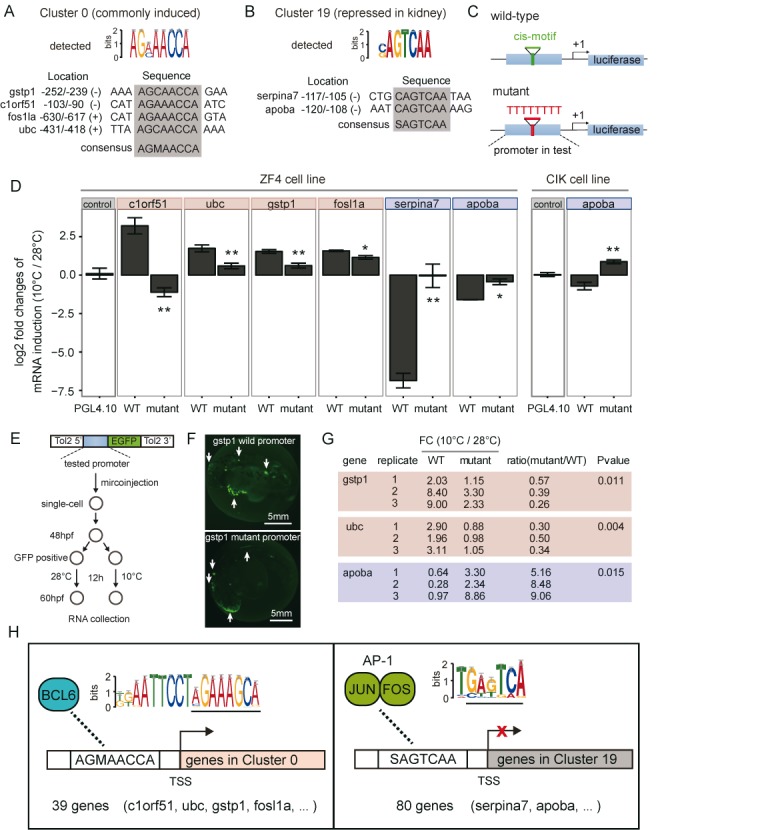
Experimental verification of the regulatory roles of two motifs, AGMAACCA and SAGTCAA, in gene expression in response to cold stress. (**A** and **B**) The locations, sequences and orientations of the *cis*-motifs within the gene promoters cloned for transfection assays. The highly conserved motif sequences in each promoter are indicated by the gray boxes. (**C**) The scheme for constructing a plasmid containing the luciferase reporter gene driven by the native promoter (top) or the motif-replaced promoter (bottom). (**D**) Ratios of reporter mRNA induction or repression at 10°C relative to 28°C were analysed using the wild-type and mutant constructs by transfecting zebrafish ZF4 and grass carp CIK cell lines. Statistically significant differences in mRNA induction or repression between the wild-type and mutant promoters are indicated by asterisks (* indicates *P* < 0.05, and ** indicates *P* < 0.01, t-test). An empty vector, PGL4.10, was used as a control. WT: wild-type. (**E**) The experimental design for examining the cold-responsive functions of the selected motifs by transgenic study of zebrafish embryos. The structure of the promoter construct and the workflow are shown. (**F**) Green fluorescence images, showing EGFP expression under the control of the wild-type (top) and mutant (bottom) *gstp1* promoters. (**G**) Relative expression levels of *egfp* in transgenic zebrafish embryos at cold (10°C) and normal (28°C) temperatures, as determined by RT-qPCR. The wild-type and mutated promoters of the same three genes (*gstp1, ubc* and *apoba*) as those used in the cell transfection experiments were examined. Fold changes were calculated by normalizing the mRNA levels at 10°C to those at 28°C. Student's t test was used to assess significant differences between the mutant/WT ratio and a value of 1. (**H**) Model of *cis*-regulation of cold-induced (red) and cold-repressed (blue) gene expression by Bcl6 and AP-1, respectively. Bcl6 positively regulates 39 genes from Cluster 0 via the AGMAACCA *cis*-motif, whereas AP-1 down-regulates the expression of 80 genes via the SAGTCAA motif. The known binding sites for Bcl6 and AP-1 are indicated at the top of each panel. The region that was predicted to contain the enriched motif by DREME is underlined.

Among the enriched motifs, we selected 2 representative motifs for experimental validation, including the novel motif AGMAACCA, which was identified in the genes in the most populated cluster (Cluster 0, with 231 genes) that were commonly induced, and SAGTCAA, which is a known AP-1 binding site that was detected in the genes in Cluster 19, which showed decreased expression in the kidney under cold stress. We selected the following four genes harbouring the AGMAACCA motif that are involved in diverse biological processes: *ubc*, which participates in proteolysis; *fosl1a*, which is a stress-related transcription factor; *gstp1*, which is known to be associated with the oxidative stress response; and *c1orf51*, which is a novel predicted gene (Figure 5A). For the SAGTCAA motif, the motif-bearing genes *serpina7* and *apoba* were chosen (Figure 5B).

The firefly luciferase reporter system was used to verify the regulatory abilities of the motifs under cold stress. ∼400–1000 bp of the promoter regions of the chosen genes containing either the wild-type motif or an oligo, ‘TTTTTTTTT’, which replaced this motif, were fused to a firefly luciferase reporter gene (Figure 5C). The constructs were transfected into cultured zebrafish ZF4 cells and incubated at 28 or 10°C. The cells were assayed for promoter activity at the two temperatures. We quantified the transcriptional changes of the reporter gene under cold stress by normalizing its transcription level at 10°C to that at the control temperature of 28°C. We observed that the wild-type promoters of all four genes containing the AGMAACCA motif were induced at 10°C, whereas the two promoters containing the SAGTCAA motif were repressed. For both motifs, the cold-induced expression changes were significantly decreased when the wild-type motifs were mutated (Figure 5D). Because the ZF4 cell line is derived from an embryonic zebrafish cell, its suitability for use in the validation of genes that are specifically repressed in the kidney, such as *apoba* in the abovementioned experiment, is questionable. However, we transferred the same *apoba* promoter constructs to cultured CIK cells, which is a cell line derived from grass carp kidney (35), and found that it showed a similar cold-repressive function in this cell line (Figure 5D).

To confirm the cold-responsive functions of the two motifs *in vivo*, the *gstp1, ubc* and *apoba* promoter sequences and the corresponding mutants were cloned into a Tol2 transposable element-based transgenic vector for microinjection into zebrafish embryos. The promoter activities at different temperatures were evaluated by measuring the expression of the target gene, *egfp*, as outlined in Figure 5E. All of the promoters were demonstrated to have function of driving gene expression in the zebrafish embryos, as exemplified by the *gstp1* promoter shown in Figure 5F. We quantified the transcriptional changes of *egfp* under cold stress by normalizing the expression level of the reporter gene at 10°C to that at the control temperature of 28°C. As expected, the *gstp1* and *ubc* promoters were induced by the cold temperature, whereas the *apoba* promoter was strongly repressed under *in vivo* conditions. When the specific AGMAACCA and SAGTCAA motifs were mutated, the levels of induction and repression by cold were greatly diminished, further confirming their cold-regulatory functions (Figure 5G). In this experiment, the *c1orf51* and *fosl1a* promoters were not examined because we found that the respective endogenous genes were not expressed in 48 hpf embryos at the normal temperature of 28°C (Supplementary Figure S7), suggesting that their expression may vary according to the developmental stage and the surrounding cellular environment (i.e., *in vivo* versus in culture).

A search against JASPAR database revealed that the SAGTCAA motif is identical to the binding site of AP-1 (Table 2). AP-1 is a transcription factor complex consisting of Fos and Jun family proteins. On the other hand, the AGMAACCA motif is highly similar to the 3′ half of the known binding site of BCL6 in humans (Figure 5H). Alignment of the human BCL6 motif with that resides in the zebrafish *c1orf51* promoter revealed additionally shared nucleotides between the two (Supplementary Figure S8). Therefore, the newly identified zebrafish motif might be equivalent to the human BCL6-binding motif. Expression analysis of transiently transfected cells showed that reporter gene expression changes under cold stress induced by the *c1orf51* promoter were particularly dramatic (Figure 5D). In addition, among the 39 co-regulated AGMAACCA motif-bearing genes in Cluster 0, at least one gene, *id2*, has been recently identified as a target of BCL6 in humans (51). Based on these findings, we propose that these two motifs have regulatory roles in the cold response in zebrafish, as shown in Figure 5H.

### The protein–protein interaction network among cold-regulated genes and transcription factors

**Figure 6.**
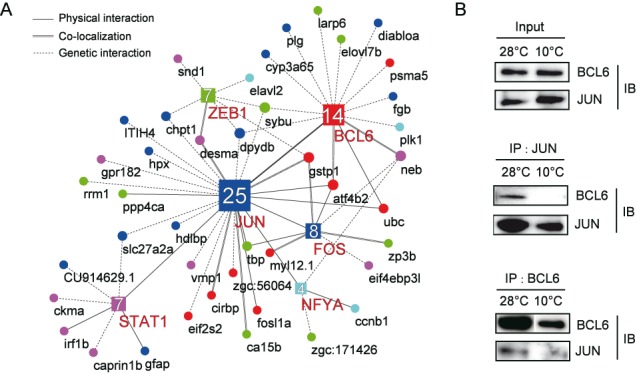
Regulatory networks, showing the protein and gene interactions among the motif-bearing genes and the binding TFs. (**A**) Circular nodes denote the classifications of the genes in the identified gene clusters, with red indicating the AGMAACCA motif in Cluster 0, blue depicting the SAGTCAA motif in Cluster 19, magenta denoting the TTCCAGBA motif in Cluster 1, green depicting the WCACCTGW motif in Cluster 7 and cyan indicating the CCAATCAG motif in Cluster 21. The quadrate nodes denote the binding TFs, with each colour indicating the TF–promoter relationship with the gene of the same colour. Protein–protein interaction information was retrieved from the literature using GeneMANIA. The types of protein–protein interactions are depicted by different types of lines, with the solid lines representing physical interactions, the parallel lines depicting co-localization, and the dots representing genetic interactions. According to the definitions in GeneMANIA, ‘physical interaction’ refers to an experimentally validated protein–protein interaction, and ‘co-localization’ refers to two proteins that co-localize in the cell. ‘Genetic interaction’ indicates that the effects of one gene are modified by another gene, as identified through mutational analysis. The number of connections associated with each TF is indicated in the quadrate nodes. Protein–protein interaction information was retrieved from the literature using GeneMANIA, and the network was constructed using Cytoscape software. (**B**) Validation of the interaction between Jun and Bcl6 by CoIP. ZF4 cells maintained at 28°C and 10°C were harvested for immunoprecipitation (IP) with a Jun or Bcl6 antibody. Input (top) and immunoprecipitates of Jun (middle panel) and Bcl6 (bottom panel) were analysed by immunoblotting (IB) with antibodies against Jun and Bcl6.

To further evaluate the roles of individual factors in the cold-responsive network, we obtained evidence of protein interactions (genetic interactions, physical interactions or co-localization) between motif-bearing genes and transcription factors (TFs). Currently, little information on protein interactions is available for zebrafish, which prompted us to use the protein–protein interaction information for humans recorded in GeneMANIA database (52). Based on the sequences of the *cis*-motifs, six TFs, Bcl6, Jun, Fos, Stat1, Nfya and Zeb1, were confidently predicted to be the *trans*-factors that bind to the *cis*-motifs and regulate their target genes during cold stress. A total of 42 genes in five gene clusters were found to have physical or genetic interactions with the six TFs. By incorporating protein interaction evidence with our data on the motif-bearing genes, we constructed a regulatory network with 61 edges (Figure 6A). Some genes that contain defined *cis*-motifs were found to interact with their binding TFs. The direct physical interaction between a TF and the protein encoded by the target gene might imply the existence of a regulatory loop between the two, which is indeed the case for the *ubc*-Bcl6 pair. The interaction of UBC with BCL6 has been reported to result in ubiquitination and degradation of BCL6 (53,54). In addition to interacting with their respective target genes, these TFs might also interact with factors outside of their defined clusters, indicating the existence of interconnected networks between the cold-responsive factors. To identify the hub TFs within the network, we summed the number of connections associated with each TF. Notably, we found that a component of the AP-1 complex, Jun, directly interacted with 4 other TFs and had the largest number (n = 25) of connections within the network (Figure 6A).

Given the importance of Jun and Bcl6 in the network, as well as the possibility of a physical interaction between these two TFs, as indicated in GeneMANIA database, we examined their potential interaction in the zebrafish/teleost system. To accomplish this goal, we co-immunoprecipitated them from ZF4 cells, clearly demonstrating their direct interaction. Moreover, the amount of the complexes was markedly reduced following cold treatment at 10°C compared with that at 28°C (Figure 6B and Supplementary Figure S9). The cold-dependent regulation mediated by these two interacting TFs would result in a cold-responsive cascade involving multiple downstream genes in the network. In summary, the constructed network expands upon the interactions between the motif-bearing genes and their associated TFs.

## DISCUSSION

### Conservation and diversification of cold-responsive gene expression in fish

To delineate the regulatory networks that underlie the cold response in zebrafish, we comprehensively examined the gene expression patterns in all major tissue types for two different low-temperature challenges using deep RNA-Seq. In terms of the number of DEGs between the normal and cold-stressed conditions, the kidney, which is involved in haematopoiesis, excretion and immune responses in teleosts, was found to have the greatest number, whereas the brain was determined to have the lowest number. Gene expression patterns under cold stress have also been investigated in seven tissues of a eurythermal fish, the common carp (*C. carpio*). A comparison of the results of that study with those of the present study revealed that the brains of these two fishes have substantially different cold responses, as determined by the number of DEGs identified in each tissue (Supplementary Figure S10A). The zebrafish brain exhibited the lowest number of DEGs among the tissues, which is the complete opposite of what was observed in the common carp. Differences in gene expression patterns were also observed between the two fish in other tissues, such as kidney and intestine. Overall, different tissues respond to cold stress with distinct strategies, and these strategies must rely heavily on background genetic programs operating in the specific tissue types. This is supported by the finding that the biological processes represented by GO terms are distributed among various clusters, which consisted of DEGs associated with different tissues or temperatures (Figure 3).

Despite the widespread tissue-specific responses, a core set of genes was induced or repressed in all or multiple tissue types. We identified 231 genes that were commonly induced and 121 genes that were commonly repressed across all 8 tissues, and the other genes showed consistent expression in seven or fewer tissues. The identification of the common cold-responsive genes in zebrafish allowed for their comparison with genes that are typically induced in the common carp during cold stress (5). Between the two fishes, 38 genes were found to be identical (Supplementary Figure S10B and Table S8). These genes are involved in oxidative phosphorylation, protein folding and degradation, RNA processing and translation, which comprise the set of evolutionarily conserved cold-responsive mechanisms in the teleost. These processes also may be active in other animal groups in response to cold stress (55).

Out of the 231 commonly induced genes, 101 have been previously reported to be induced in larval zebrafish (9,11) (Supplementary Table S8). Most of the genes are involved in the same biological processes noted above, for example, the *cox* gene family (*cox5b, cox6a1, cox7a2a* and *cox7c*) and *v-atpases* (*atp5ia, atp5ib, atp5l* and *atp5o*), which are involved in oxidative phosphorylation. However, we did observe more pronounced expression changes in antioxidant enzymes (*ptgs2a, ptgs2b, gstp1, gpx4b* and *prdx1*), several stress-related transcription factors (*hig1, nfkbiaa, fosl1a, junb, cebpb, fkbp25, klf2* and *klf11*) and 3 pro-apoptotic genes (*gadd45aa, gadd45ab* (56) and *aif* (57)) that do not belong to the evolutionarily conserved responsive gene set, suggesting species-specific differences between the common carp and zebrafish. Whether these differences translate into different levels of cold tolerance between the two species requires further investigation. In nature, common carp are more resistant to cold temperatures than zebrafish.

Due to a substantial increase in the number of genes available for multi-tissue screening (22 340 compared with 13 440 for the common carp), we identified 92 additional commonly induced genes in this study. These genes include *gpd1b*, which has been shown to accumulate in response to cold stress in yeast (58), and *atf4b2*, a transcription factor involved in regulation of the cold-adaptive response via activation of downstream targets associated with energy homeostasis in hibernating ground squirrels (38) and mice (39). These factors may belong to the evolutionarily conserved regulatory networks that have been overlooked in previous studies due to the limited sequencing technologies available at the time.

Similar to the commonly induced genes, the commonly repressed genes included many key genes involved in the oxidation-reduction process (for example, *cyp2n13, cyp1c1* and *faxdc2*), indicating the overall slowing of biological reactions at cold temperatures (Figure 3). Repression of oxidation-related genes would reduce the mitochondrial respiratory rate and thus minimize oxidative stress in cold temperatures (7,59).

### Evolution of *cis*-regulatory elements in the cold-responsive network of the fishes

The integration of multi-tissue transcriptional changes into a definitive set of genetic circuits is highly desirable but remains challenging in the study of teleost cold responses. By examining zebrafish cold-induced expression patterns with motif identification tools, we identified distinct *cis*-regulatory elements in the promoters of co-regulated genes that likely participate in regulatory networks in zebrafish. This is the first report of the use of this type of approach to study the teleost cold response, and it was shown to be valid and effective.

We identified a few *cis*-regulatory motifs that are known to be cold responsive in other animals, such as the AP-1 binding motif and the Stat1 binding site, confirming the validity of this approach. More notably, we were able to pinpoint motifs that are not known to be cold responsive. For example, the motif AGMAACCA was over-represented in 17% of the commonly induced genes and was predominantly found in genes annotated with the ‘response to cold’ GO classification (Supplementary Table S5). Cell transfection and *in vivo* transgenic analysis with reporter genes revealed that this motif is an authentic cold-responsive element involved in transcriptional regulation. We identified a series of such elements (Table 2) and have provided data for further in-depth studies.

The identification of potential cold-responsive regulatory motifs paved the way for identifying the *trans*-regulatory factors involved in the cold response. We identified a series of *cis*-motifs and their binding *trans*-factors, such as the AGMAACA motif for Bcl6 binding and the SAGTTC motif for AP-1 binding. The elucidation of this genetic network provides new insights into the molecular mechanisms underlying cold adaptation in fishes. To gain insight into the degree of conservation of this network in fishes, we examined the presence of the Bcl6 and AP-1 binding sites in regions 1 kb upstream of orthologous genes in four other fish species that have adapted to different thermal habitats. We found that the number of genes bearing these motifs varied among the species and that generally, the sub-Arctic and temperate fishes (Atlantic cod, common carp and pufferfish) have fewer genes with these two motifs in the 1 kb upstream regions, while the two tropical fishes, tilapia and zebrafish, have as many as 47 and 119 genes, respectively, containing them (Supplementary Figure S11). Notably, some orthologous genes do not completely correspond between zebrafish and the other four fish species evaluated due to the loss or gain of genes among these species. Nonetheless, we observed the global dynamic patterns in the promoters of these orthologous genes. Our results indicate that the regulatory network for the cold response is markedly different among fish species and that these differences may be influenced by species’ evolutionary histories. The regulatory network identified in our study may serve as a foundation for investigating the evolution of cold-responsive elements in various fish species.

In summary, by integrating the transcriptional profiling data with the annotated genome, we successfully delineated several genetic networks regulating the responses of zebrafish to mild and severe cold. The newly identified *cis-* and *trans-*regulatory factors provide new insights into the molecular mechanisms of cold responses and thermal adaptation in teleost fishes. Due to the limited upstream regions in which the motifs were searched for and the inadequate sensitivities of the detection programs, the current list of motifs is probably not complete. Further studies with shorter sampling intervals and programs that are more amenable to searching a larger area may improve the completeness and accuracy of the network. Nonetheless, the discovery of novel cold-responsive motifs and their associated TFs in this work suggests that our approach is promising. Such a systematic approach could be useful in broader studies of environmental genomics.

## DATA AVAILABILITY

The sequencing data have been deposited in GEO at NCBI under the accession number GSE62221.

## Supplementary Material

SUPPLEMENTARY DATA
