# Supplementary material for: Global identification of the genetic networks and cis-regulatory elements of the cold response in zebrafish
Source: Nucleic Acids Res. 2015 Jul 30;43(19):9198–213. doi: 10.1093/nar/gkv780 (PMC4627065; doi:10.1093/nar/gkv780)
Supplement: SUPPLEMENTARY DATA [file supp_43_19_9198__index.html]

Global identification of the genetic networks and cis-regulatory elements of the cold response in zebrafish — Global identification of the genetic networks and cis-regulatory elements of the cold response in zebrafish — SUPPLEMENTARY DATA 

# Global identification of the genetic networks and *cis*-regulatory elements of the cold response in zebrafish

## SUPPLEMENTARY DATA

- SUPPLEMENTARY DATA
- SUPPLEMENTARY DATA
