# Supplementary material for: Global identification of the genetic networks and cis-regulatory elements of the cold response in zebrafish
Source: Nucleic Acids Res. 2015 Jul 30;43(19):9198–213. doi: 10.1093/nar/gkv780 (PMC4627065; doi:10.1093/nar/gkv780)
Supplement: SUPPLEMENTARY DATA [file supp_gkv780_nar-03715-z-2014-File008.pdf]

## SUPPLEMENTARY DATA

**Table S1: List of primers used in this study.**

**Table S2: The numbers of raw, clean and mapped RNA-Seq reads for each sample.**

**Table S3: List of differentially expressed genes in zebrafish between the normal and cold temperatures.**

**Table S4: List of genes and their cold-responsive expression patterns for clusters obtained by fuzzy K-means clustering.**

**Table S5: Enriched GO terms in each cluster, along with the –log10 transformed p value for each GO term identified in enrichment analysis.**

**Table S6: List of genes with enriched cis-motif(s).**

**Table S7: Percentage of genes with certain cis-motifs among the gene set associated with the enriched GO terms shown in Figure 4.**

**Table S8: Breakdown of the Cluster 0 (commonly induced) genes into three categories: 'fish core set', 'zebrafish core set' and 'previously undescribed set'.**

**Figure S1: Electropherograms of total RNAs from 24 samples.**

**Figure S2: Electropherograms of cDNA libraries of 24 samples.**

**Figure S3: Characterization of gene expression patterns in zebrafish tissues under cold stress.** (A). Venn diagrams showing the distribution of genes with > 2-fold changes in expression among the three comparisons. (B). The relative expression changes of the DEGs at 18°C and 10°C after normalization to 28°C in the kidney. (C). Sampling strategy for examining the effect of prolonged 18°C exposure on gene expression patterns in zebrafish. Zebrafish reared at and acclimated to the normal temperature (28°C) were gradually cooled to 18°C using the same procedure as that described in the previous experiment, and the fish were maintained at 18°C for 36 h. The sampling time points are denoted by three dots, with the letters a, b and c (a-normal 28°C; b-18°C, 12 h; and c-18°C, 36 h). (D) Scatter plot showing the expression changes of 5 genes in two comparisons, namely b/a (18°C, 1 2 h/28°C) and c/b (18°C, 36 h/18°C 12 h), in multiple tissues. Two micrograms of RNA from each individual tissue sampled at one of the three time points was reverse transcribed. An equal amount of the resultant cDNA product was subjected to quantitative PCR for amplification of the 5 genes that showed significant cold-responsive transcriptional activity in our previous experiment. The relative

expression level of each gene in each tissue was calculated for the two comparisons, and the ratio was $log_2$ transformed and plotted. The X axis indicates the change in expression deduced from c/b, and the Y axis represents that from b/a. The distribution of the genes in the x-y space clearly indicates greater expression changes for the 18°C, 12 h/28°C comparison compared with the 18°C, 36 h/18°C 12 h comparison. The different genes and tissues examined are indicated by the different shapes and colours.

**Figure S4: The expression patterns of 12 genes under cold treatments, as measured by RT-qPCR, compared with those deduced from the RNA-Seq results.**

**Figure S5: Heatmap showing the clustering results of the 1943 differentially expressed genes.**

**Figure S6: Sequence alignments between the enriched cis-motifs and the matched entries in JASPAR database.**

**Figure S7: Agarose gel electrophoresis, showing the RT-PCR products of 4 endogenous genes in zebrafish embryos (48 hpf) maintained at 28°C or exposed to 10°C for 12 h.** The results suggest lack of expression of the *c1orf51* and *fos1la* genes in the embryos at 28°C. Because of this lack of expression, it was impossible to assess the effect of motif mutations on gene expression at 28°C. These two genes were not selected for the *in vivo* transgenic study.

**Figure S8: Sequence alignment of the AGMAACCA motif, the Bcl6 binding site and the corresponding sequences in the four validated promoters.** The identical bases are indicated by triangles, and the number of identical bases is indicated on the right.

**Figure S9: Validation of the protein interaction between Jun and Bcl6 by CoIP in larger images, with the size markers shown.** ZF4 cells at 28°C and 10°C were harvested for immunoprecipitation (IP) with a Jun or Bcl6 antibody. Input and immunoprecipitates were analysed by immunoblotting (IB) with antibodies against Bcl6 (A) and Jun (B). The target band is indicated by red arrows in the blot. PageRuler Prestained Protein Ladder was used as a protein ladder (#26616, Thermo).

**Figure S10: Comparison of the cold-responsive patterns between zebrafish and common carp.** (A). The distributions of the numbers of differentially expressed genes among the tissues under cold stress in zebrafish and common carp. Red indicates up-regulated genes, and blue indicates down-regulated genes. (B) Heatmap for the gene sets listed in Table S6. The ‘fish core set’ was deduced by a comparison with commonly induced genes from common carp. The ‘zebrafish core set’

contains genes that have been reported to be up-regulated in larval zebrafish. The 'previously undescribed set' contains genes that have not been previously reported.

**Figure S11: Analysis of the presence of the Bcl6 and AP-1 binding sites in upstream 1 kb regions of zebrafish orthologous genes from other fish species.** Four fish species, including Atlantic cod (*Gadus morhua*), common carp (*Cyprinus carpio*), pufferfish (*Tetraodon nigroviridis*) and tilapia (*Oreochromis niloticus*), were chosen for analysis. For zebrafish, 39 commonly induced genes containing the Bcl6 binding motif in their promoters and 80 kidney-repressed genes containing the AP-1 binding motif in their promoters were included in analysis. The genes are listed in Supplementary Table S6. For Atlantic cod, pufferfish and tilapia, the orthologous gene pairs between zebrafish and each of the three fish species were obtained from Biomart in Ensembl. We obtained 94, 89 and 116 orthologous genes for Atlantic cod, pufferfish and tilapia, respectively. We retrieved the corresponding Uniprot IDs for the common carp genes from Carpbase (common carp genome database, http://www.carpbase.org/). Then, we retrieved the Uniprot IDs for the zebrafish genes from Ensembl. The zebrafish-common carp orthologous gene relationships were referenced using the same Uniprot IDs. We identified 116 orthologous genes in common carp. The genome versions used in the study were as follows: Atlantic cod (gadMor1), common carp (GCA_000951615.1), pufferfish (TETRAODON8.0), tilapia (Orenil1.0), and zebrafish (ZV9). The 1 kb upstream sequences of the orthologous genes were retrieved from their respective genomes based on the gff file, which denoted the coordinates of the genes within chromosomes or scaffolds. FIMO motif scanning software in MEME Suite was used to search for the Bcl6 and AP-1 binding motifs in the promoter sequences. Promoter sequences with a p value of less than the default threshold (0.0001) were considered to contain a binding motif. The number of the genes with the Bcl6 and AP-1 binding motifs were counted for each species.

# Figure S1

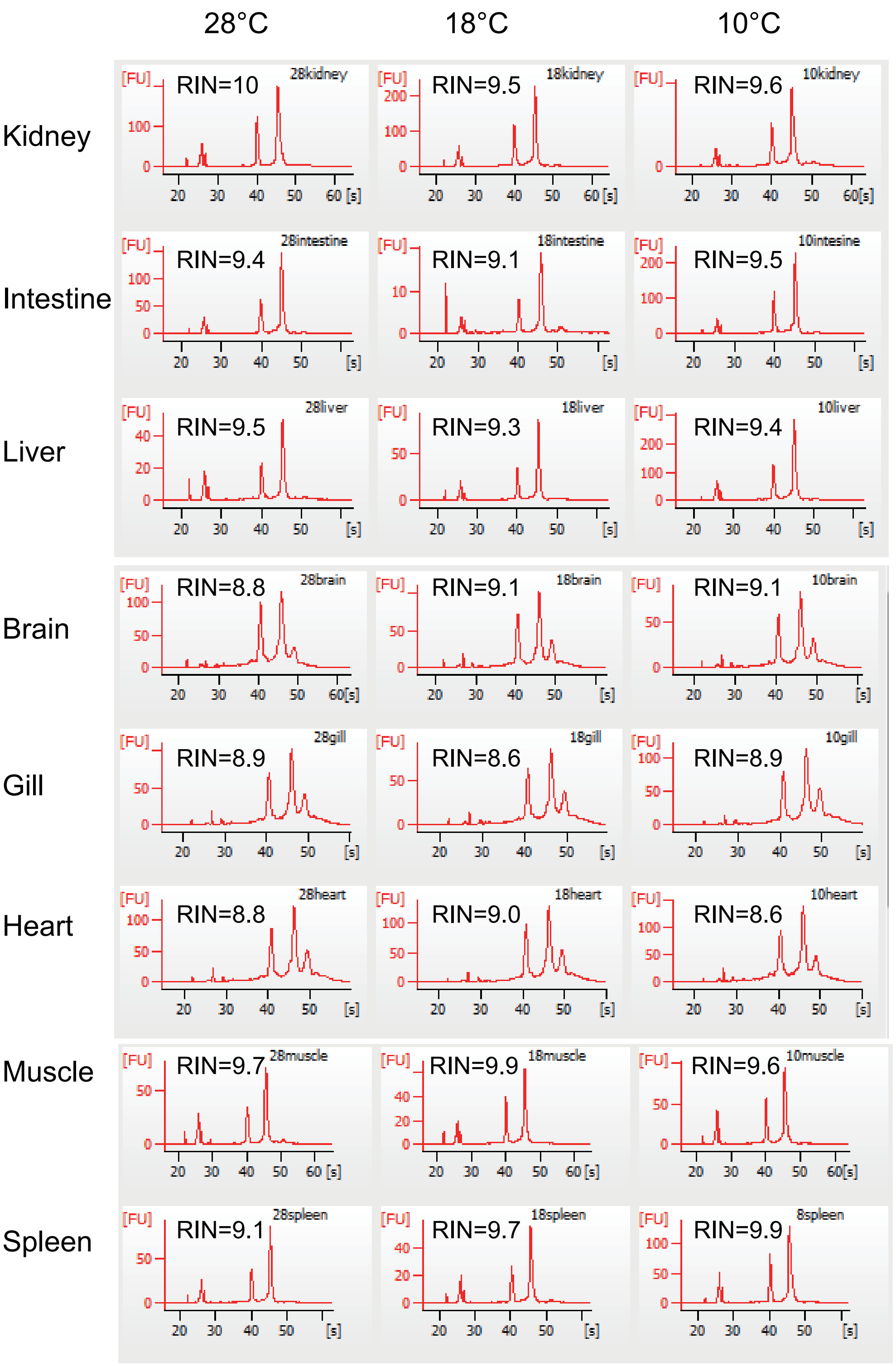
28°C
18°C
10°C
Kidney
Intestine
Liver
Brain
Gill
Heart
Muscle
Spleen
RIN=10
28kidney
RIN=9.5
18kidney
RIN=9.6
10kidney
RIN=9.4
28intestine
RIN=9.1
18intestine
RIN=9.5
10intesine
RIN=9.5
28liver
RIN=9.3
18liver
RIN=9.4
10liver
RIN=8.8
28brain
RIN=9.1
18brain
RIN=9.1
10brain
RIN=8.9
28gill
RIN=8.6
18gill
RIN=8.9
10gill
RIN=8.8
28heart
RIN=9.0
18heart
RIN=8.6
10heart
RIN=9.7
28muscle
RIN=9.9
18muscle
RIN=9.6
10muscle
RIN=9.1
28spleen
RIN=9.7
18spleen
RIN=9.9
8spleen
[FU]
[s]

# Figure S2

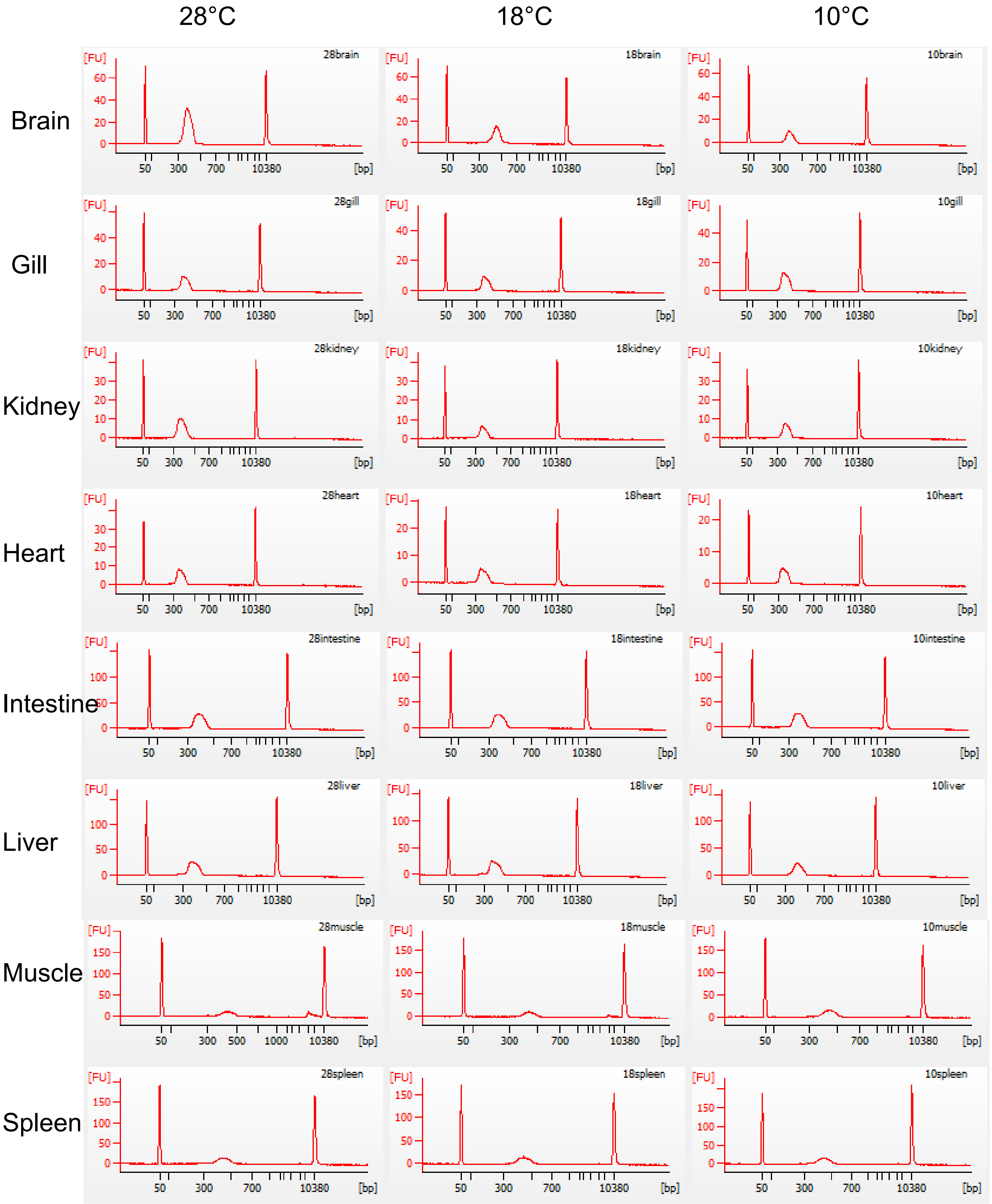

28°C
18°C
10°C
Brain
Gill
Kidney
Heart
Intestine
Liver
Muscle
Spleen
28brain
18brain
10brain
28gill
18gill
10gill
28kidney
18kidney
10kidney
28heart
18heart
10heart
28intestine
18intestine
10intestine
28liver
18liver
10liver
28muscle
18muscle
10muscle
28spleen
18spleen
10spleen
[FU]
[bp]
50
300
500
700
1000
10380

# Figure S3

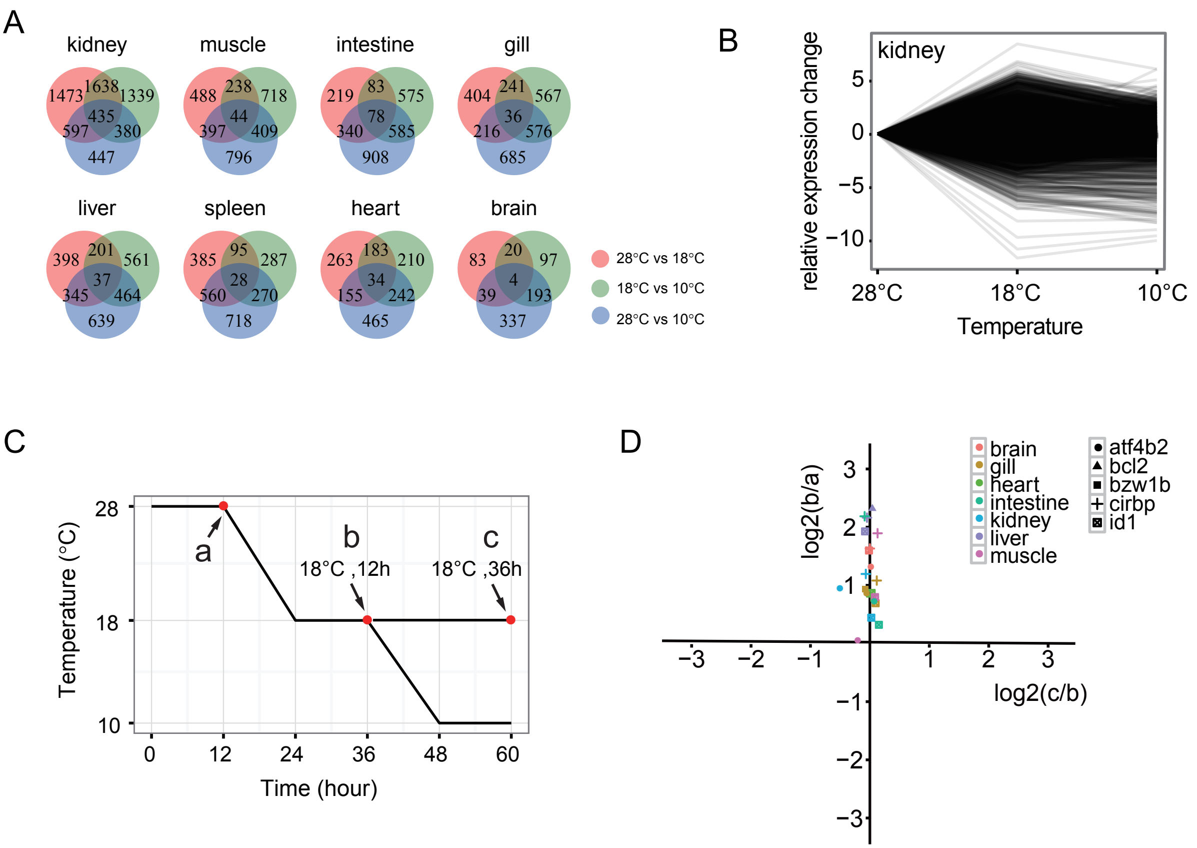

A
kidney
1473
1638
1339
435
597
380
447
muscle
488
238
718
44
397
409
796
intestine
219
83
575
78
340
585
908
gill
404
241
567
36
216
576
685
liver
398
201
561
37
345
464
639
spleen
385
95
287
28
560
270
718
heart
263
183
210
34
155
242
465
brain
83
20
97
4
39
193
337
28°C vs 18°C
18°C vs 10°C
28°C vs 10°C
B
kidney
relative expression change
5
0
−5
−10
28°C
18°C
10°C
Temperature
C
Temperature (°C)
28
18
10
a
b
18°C ,12h
c
18°C ,36h
0
12
24
36
48
60
Time (hour)
D
log2(b/a)
log2(c/b)
brain
gill
heart
intestine
kidney
liver
muscle
atf4b2
bcl2
bzw1b
cirbp
id1

# Figure S4

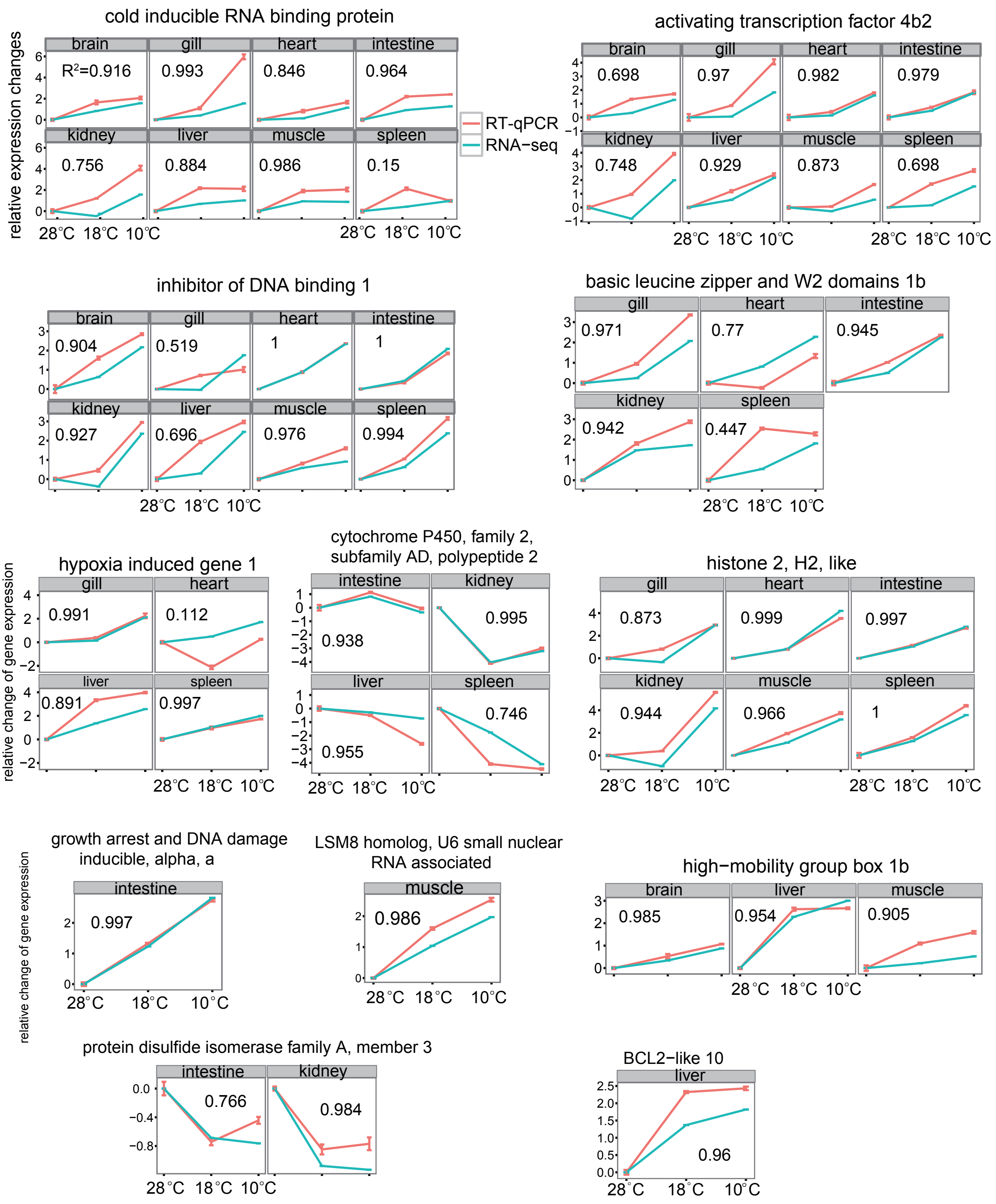

cold inducible RNA binding protein
brain
gill
heart
intestine
kidney
liver
muscle
spleen
$R^2$=0.916
0.993
0.846
0.964
0.756
0.884
0.986
0.15
relative expression changes
RT−qPCR
RNA−seq
28°C 18°C 10°C
activating transcription factor 4b2
0.698
0.97
0.982
0.979
0.748
0.929
0.873
0.698
inhibitor of DNA binding 1
0.904
0.519
1
1
0.927
0.696
0.976
0.994
basic leucine zipper and W2 domains 1b
0.971
0.77
0.945
0.942
0.447
hypoxia induced gene 1
0.991
0.112
0.891
0.997
relative change of gene expression
cytochrome P450, family 2, subfamily AD, polypeptide 2
0.938
0.995
0.955
0.746
histone 2, H2, like
0.873
0.999
0.997
0.944
0.966
1
growth arrest and DNA damage inducible, alpha, a
0.997
LSM8 homolog, U6 small nuclear RNA associated
0.986
high−mobility group box 1b
0.985
0.954
0.905
protein disulfide isomerase family A, member 3
0.766
0.984
BCL2−like 10
0.96

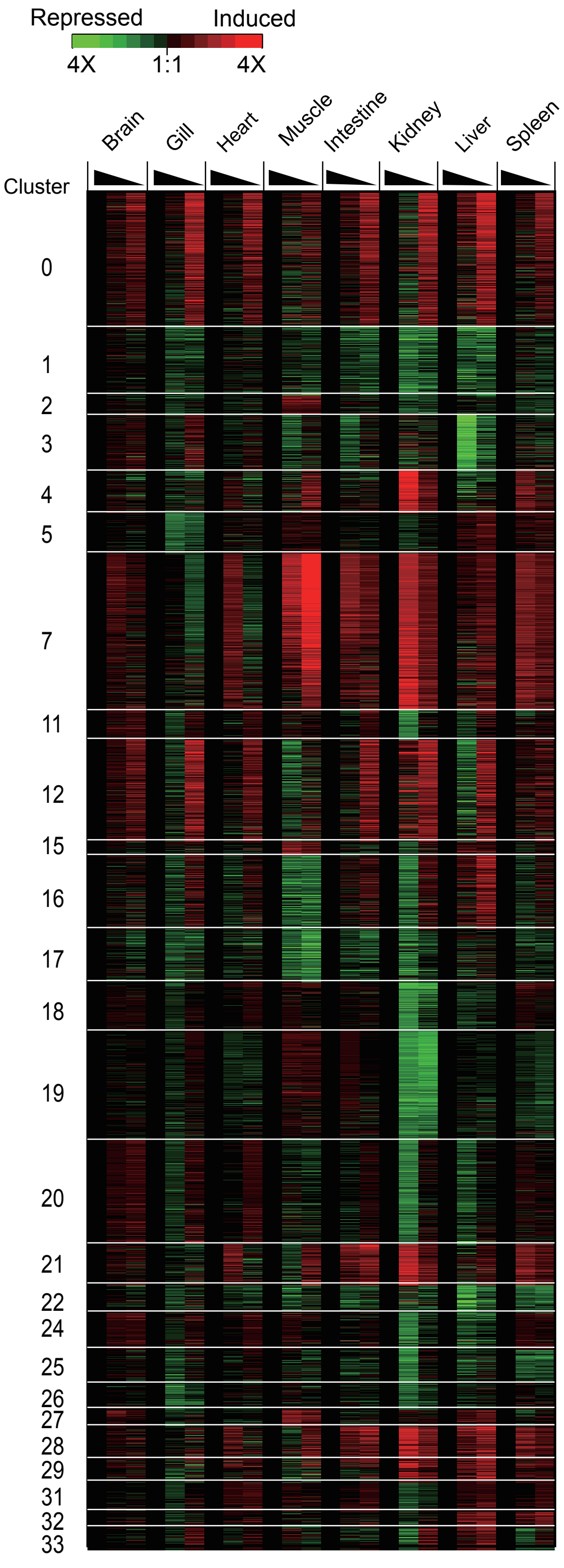
Repressed
Induced
4X
1:1
4X
Brain
Gill
Heart
Muscle
Intestine
Kidney
Liver
Spleen
Cluster
0
1
2
3
4
5
7
11
12
15
16
17
18
19
20
21
22
24
25
26
27
28
29
31
32
33

Figure S5

Figure S6

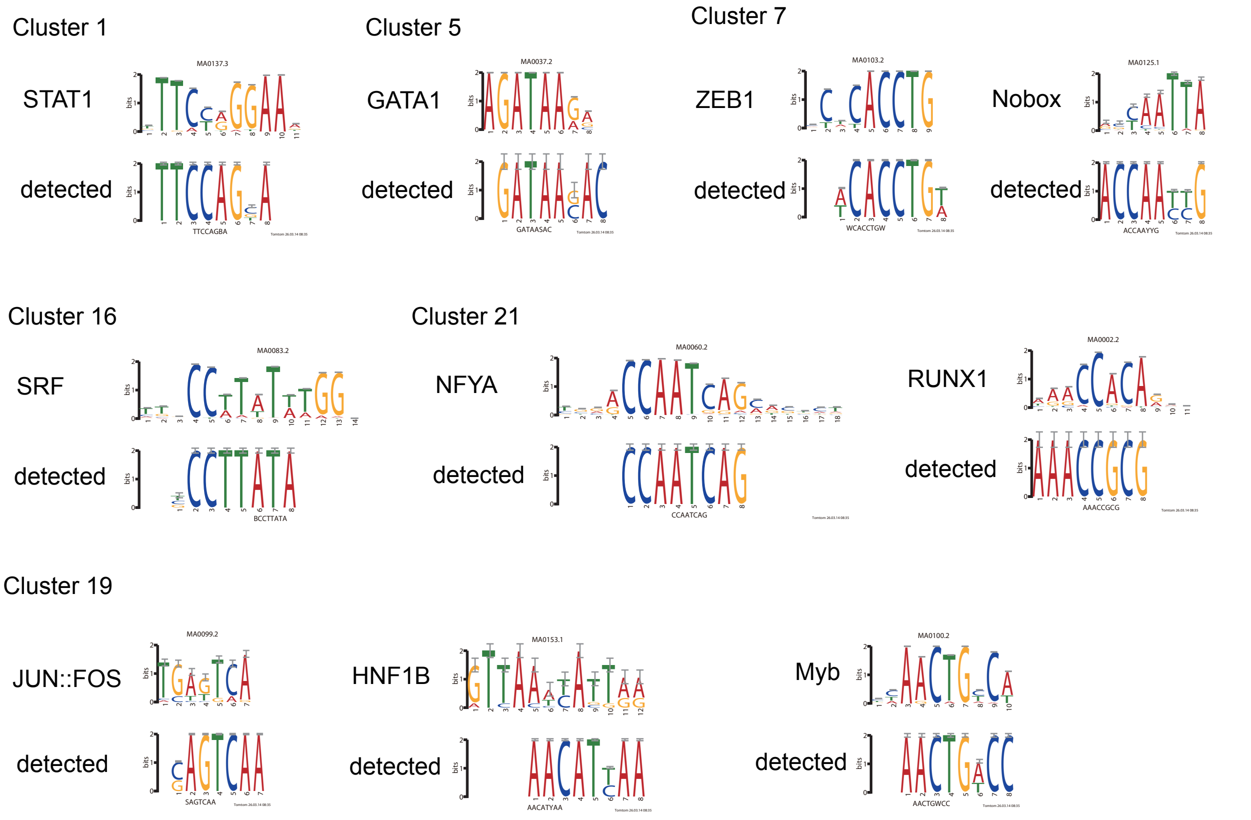
Cluster 1
STAT1
MA0137.3
detected
TTCCAGBA
Cluster 5
GATA1
MA0037.2
detected
GATAASAC
Cluster 7
ZEB1
MA0103.2
detected
WCACCTGW
Nobox
MA0125.1
detected
ACCAAYYG
Cluster 16
SRF
MA0083.2
detected
BCCTTATA
Cluster 21
NFYA
MA0060.2
detected
CCAATCAG
RUNX1
MA0002.2
detected
AAACCGCG
Cluster 19
JUN::FOS
MA0099.2
detected
SAGTCAA
HNF1B
MA0153.1
detected
AACATYAA
Myb
MA0100.2
detected
AACTGWCC

Figure S7

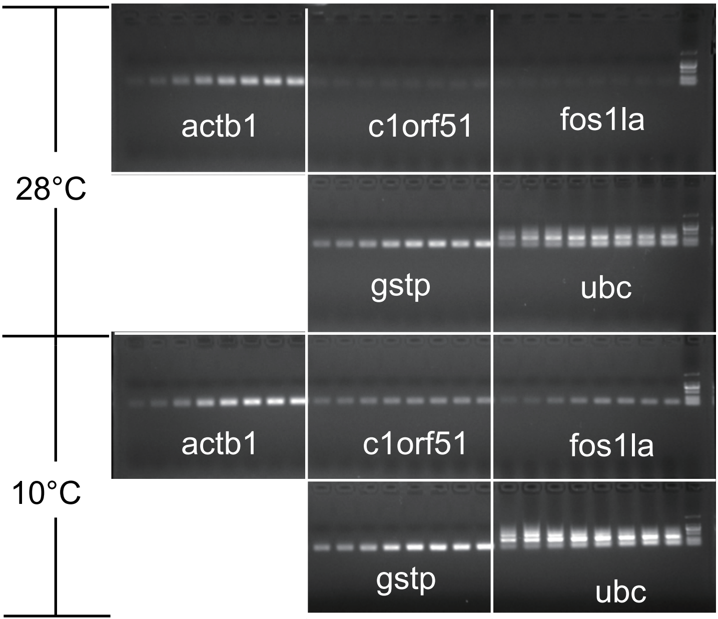

28°C
10°C
actb1
c1orf51
fos1la
gstp
ubc
actb1
c1orf51
fos1la
gstp
ubc

Figure S8

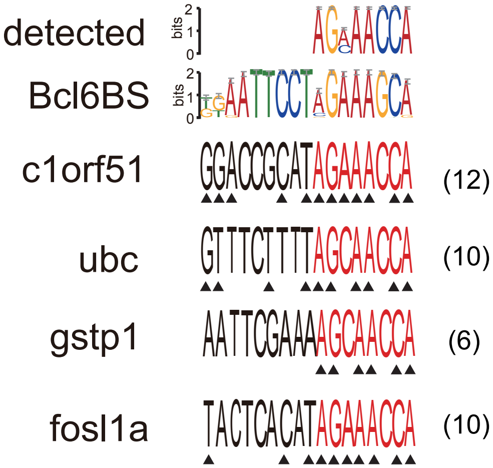
detected
Bcl6BS
bits
2
1
0
c1orf51
GGACCGCATAGAAACCA
(12)
ubc
GTTTCTTTTAGCAACCA
(10)
gstp1
AATTCGAAAAGCAACCA
(6)
fosl1a
TACTCACATAGAAACCA
(10)

Figure S9

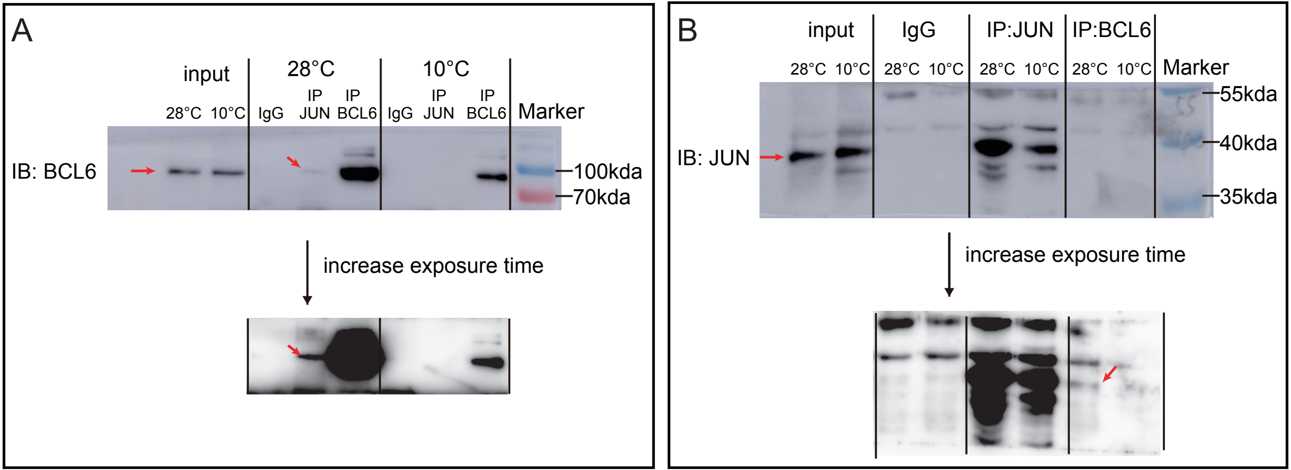
A
input
28°C
10°C
28°C
10°C
IgG
IP JUN
IP BCL6
IgG
IP JUN
IP BCL6
Marker
IB: BCL6
100kda
70kda
increase exposure time
B
input
IgG
IP:JUN
IP:BCL6
28°C
10°C
28°C
10°C
28°C
10°C
28°C
10°C
Marker
IB: JUN
55kda
40kda
35kda
increase exposure time

Figure S10

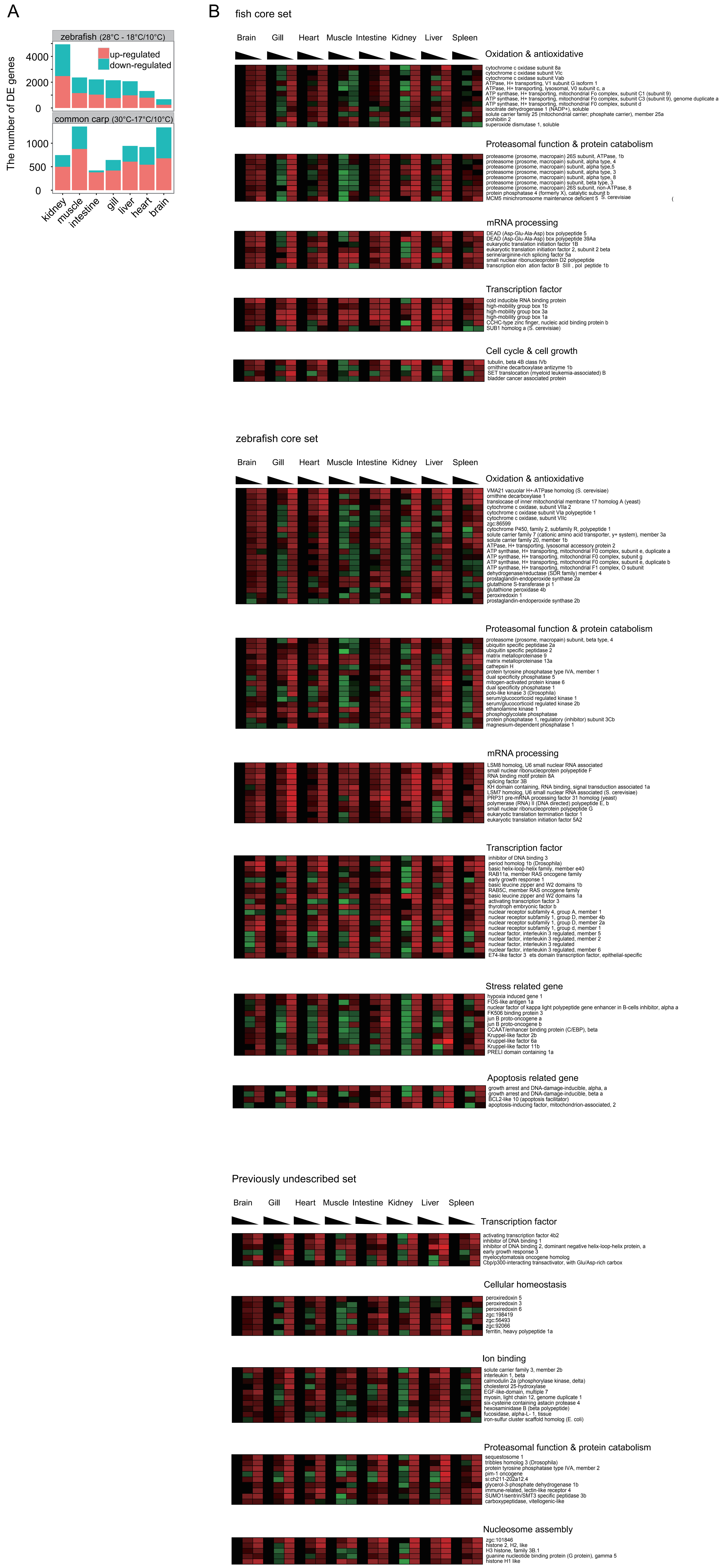

A
B
zebrafish (28°C - 18°C/10°C)
up-regulated
down-regulated
The number of DE genes
4000
2000
0
common carp (30°C-17°C/10°C)
1000
500
0
kidney
muscle
intestine
gill
liver
heart
brain
fish core set
Brain
Gill
Heart
Muscle
Intestine
Kidney
Liver
Spleen
Oxidation & antioxidative
cytochrome c oxidase subunit 8a
cytochrome c oxidase subunit VIc
cytochrome c oxidase subunit Vab
ATPase, H+ transporting, V1 subunit G isoform 1
ATPase, H+ transporting, lysosomal, V0 subunit c, a
ATP synthase, H+ transporting, mitochondrial Fo complex, subunit C1 (subunit 9)
ATP synthase, H+ transporting, mitochondrial Fo complex, subunit C3 (subunit 9), genome duplicate a
ATP synthase, H+ transporting, mitochondrial F0 complex, subunit d
isocitrate dehydrogenase 1 (NADP+), soluble
solute carrier family 25 (mitochondrial carrier; phosphate carrier), member 25a
prohibitin 2
superoxide dismutase 1, soluble
Proteasomal function & protein catabolism
proteasome (prosome, macropain) 26S subunit, ATPase, 1b
proteasome (prosome, macropain) subunit, alpha type, 4
proteasome (prosome, macropain) subunit, alpha type,5
proteasome (prosome, macropain) subunit, alpha type, 3
proteasome (prosome, macropain) subunit, alpha type, 8
proteasome (prosome, macropain) subunit, beta type, 3
proteasome (prosome, macropain) 26S subunit, non-ATPase, 8
protein phosphatase 4 (formerly X), catalytic subunit b
MCM5 minichromosome maintenance deficient 5 S. cerevisiae
mRNA processing
DEAD (Asp-Glu-Ala-Asp) box polypeptide 5
DEAD (Asp-Glu-Ala-Asp) box polypeptide 39Aa
eukaryotic translation initiation factor 1B
eukaryotic translation initiation factor 2, subunit 2 beta
serine/arginine-rich splicing factor 5a
small nuclear ribonucleoprotein D2 polypeptide
transcription elon ation factor B SIII , pol peptide 1b
Transcription factor
cold inducible RNA binding protein
high-mobility group box 1b
high-mobility group box 3a
high-mobility group box 1a
CCHC-type zinc finger, nucleic acid binding protein b
SUB1 homolog a (S. cerevisiae)
Cell cycle & cell growth
tubulin, beta 4B class IVb
ornithine decarboxylase antizyme 1b
SET translocation (myeloid leukemia-associated) B
bladder cancer associated protein
zebrafish core set
Brain
Gill
Heart
Muscle
Intestine
Kidney
Liver
Spleen
Oxidation & antioxidative
VMA21 vacuolar H+-ATPase homolog (S. cerevisiae)
ornithine decarboxylase 1
translocase of inner mitochondrial membrane 17 homolog A (yeast)
cytochrome c oxidase, subunit VIIa 2
cytochrome c oxidase subunit VIa polypeptide 1
cytochrome c oxidase, subunit VIIc
zgc:86599
cytochrome P450, family 2, subfamily R, polypeptide 1
solute carrier family 7 (cationic amino acid transporter, y+ system), member 3a
solute carrier family 20, member 1b
ATPase, H+ transporting, lysosomal accessory protein 2
ATP synthase, H+ transporting, mitochondrial F0 complex, subunit e, duplicate a
ATP synthase, H+ transporting, mitochondrial F0 complex, subunit g
ATP synthase, H+ transporting, mitochondrial F0 complex, subunit e, duplicate b
ATP synthase, H+ transporting, mitochondrial F1 complex, O subunit
dehydrogenase/reductase (SDR family) member 4
prostaglandin-endoperoxide synthase 2a
glutathione S-transferase pi 1
glutathione peroxidase 4b
peroxiredoxin 1
prostaglandin-endoperoxide synthase 2b
Proteasomal function & protein catabolism
proteasome (prosome, macropain) subunit, beta type, 4
ubiquitin specific peptidase 2a
ubiquitin specific peptidase 2
matrix metalloproteinase 9
matrix metalloproteinase 13a
cathepsin H
protein tyrosine phosphatase type IVA, member 1
dual specificity phosphatase 5
mitogen-activated protein kinase 6
dual specificity phosphatase 1
polo-like kinase 3 (Drosophila)
serum/glucocorticoid regulated kinase 1
serum/glucocorticoid regulated kinase 2b
ethanolamine kinase 1
phosphoglycolate phosphatase
protein phosphatase 1, regulatory (inhibitor) subunit 3Cb
magnesium-dependent phosphatase 1
mRNA processing
LSM8 homolog, U6 small nuclear RNA associated
small nuclear ribonucleoprotein polypeptide F
RNA binding motif protein 8A
splicing factor 3B
KH domain containing, RNA binding, signal transduction associated 1a
LSM7 homolog, U6 small nuclear RNA associated (S. cerevisiae)
PRP31 pre-mRNA processing factor 31 homolog (yeast)
polymerase (RNA) II (DNA directed) polypeptide E, b
small nuclear ribonucleoprotein polypeptide G
eukaryotic translation termination factor 1
eukaryotic translation initiation factor 5A2
Transcription factor
inhibitor of DNA binding 3
period homolog 1b (Drosophila)
basic helix-loop-helix family, member e40
RAB11a, member RAS oncogene family
early growth response 1
basic leucine zipper and W2 domains 1b
RAB5C, member RAS oncogene family
basic leucine zipper and W2 domains 1a
activating transcription factor 3
thyrotroph embryonic factor b
nuclear receptor subfamily 4, group A, member 1
nuclear receptor subfamily 1, group D, member 4b
nuclear receptor subfamily 1, group D, member 2a
nuclear receptor subfamily 1, group D, member 1
nuclear factor, interleukin 3 regulated, member 5
nuclear factor, interleukin 3 regulated, member 2
nuclear factor, interleukin 3 regulated
nuclear factor, interleukin 3 regulated, member 6
E74-like factor 3 ets domain transcription factor, epithelial-specific
Stress related gene
hypoxia induced gene 1
FOS-like antigen 1a
nuclear factor of kappa light polypeptide gene enhancer in B-cells inhibitor, alpha a
FK506 binding protein 3
jun B proto-oncogene a
jun B proto-oncogene b
CCAAT/enhancer binding protein (C/EBP), beta
Kruppel-like factor 2b
Kruppel-like factor 6a
Kruppel-like factor 11b
PRELI domain containing 1a
Apoptosis related gene
growth arrest and DNA-damage-inducible, alpha, a
growth arrest and DNA-damage-inducible, beta a
BCL2-like 10 (apoptosis facilitator)
apoptosis-inducing factor, mitochondrion-associated, 2
Previously undescribed set
Brain
Gill
Heart
Muscle
Intestine
Kidney
Liver
Spleen
Transcription factor
activating transcription factor 4b2
inhibitor of DNA binding 1
inhibitor of DNA binding 2, dominant negative helix-loop-helix protein, a
early growth response 3
myelocytomatosis oncogene homolog
Cbp/p300-interacting transactivator, with Glu/Asp-rich carbox
Cellular homeostasis
peroxiredoxin 5
peroxiredoxin 3
peroxiredoxin 6
zgc:198419
zgc:56493
zgc:92066
ferritin, heavy polypeptide 1a
Ion binding
solute carrier family 3, member 2b
interleukin 1, beta
calmodulin 2a (phosphorylase kinase, delta)
cholesterol 25-hydroxylase
EGF-like-domain, multiple 7
myosin, light chain 12, genome duplicate 1
six-cysteine containing astacin protease 4
hexosaminidase B (beta polypeptide)
fucosidase, alpha-L- 1, tissue
iron-sulfur cluster scaffold homolog (E. coli)
Proteasomal function & protein catabolism
sequestosome 1
tribbles homolog 3 (Drosophila)
protein tyrosine phosphatase type IVA, member 2
pim-1 oncogene
si:ch211-202a12.4
glycerol-3-phosphate dehydrogenase 1b
immune-related, lectin-like receptor 4
SUMO1/sentrin/SMT3 specific peptidase 3b
carboxypeptidase, vitellogenic-like
Nucleosome assembly
zgc:101846
histone 2, H2, like
H3 histone, family 3B.1
guanine nucleotide binding protein (G protein), gamma 5
histone H1 like

# Figure S11

| Category | Latitude | Species | # motif containing genes / orthologus | | |
|---|---|---|---|---|---|
| | | | BCL6 | AP1(JUN::FOS) | Total |
| Sub-Arctic | 83°N - 35°N | Atlantic cod (*Gadus morhua*) 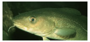 | 9/31 | 17/63 | 26/94 |
| Temperate | 60°N - 40°N | Common carp (*Cyprinus carpio*) 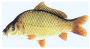 | 9/38 | 17/51 | 26/89 |
| Temperate | 44°N - 40°S | Pufferfish (*Tetraodon nigroviridis*) 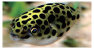 | 12/32 | 14/57 | 26/89 |
| Tropical | 32°N - 10°N | Tilapia (*Oreochromis niloticus*) 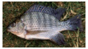 | 11/31 | 36/85 | 47/116 |
| Tropical | 33°N - 8°N | Zebrafish (*Danio rerio*) 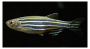 | 39/39 | 80/80 | 119/119 |
